# Altered cytoskeletal status in the transition from proneural to mesenchymal glioblastoma subtypes

**DOI:** 10.1038/s41598-022-14063-7

**Published:** 2022-06-14

**Authors:** Maureen Keller, Magdalena Blom, Lei Liu Conze, Min Guo, Daniel Hägerstrand, Pontus Aspenström

**Affiliations:** 1 Department of Immunology, Genetics and Pathology (IGP), Rudbeck Laboratory, Uppsala University, 751 85 Uppsala, Sweden; 2 Department of Microbiology, Tumor and Cell Biology, Karolinska Institutet, Stockholm, Sweden; 3 Department of Oncology and Pathology, Karolinska Institutet, Stockholm, Sweden; 4 Department of Radiology, Beijing Tiantan Hospital, Capital Medical University, Beijing, China; 5 Department of Molecular Medicine and Surgery, Karolinska Institutet, Stockholm, Sweden

**Keywords:** Tumour biomarkers, Cell migration, Cellular imaging, Cytoskeleton

## Abstract

Glioblastoma is a highly aggressive brain tumor with poor patient prognosis. Treatment outcomes remain limited, partly due to intratumoral heterogeneity and the invasive nature of the tumors. Glioblastoma cells invade and spread into the surrounding brain tissue, and even between hemispheres, thus hampering complete surgical resection. This invasive motility can arise through altered properties of the cytoskeleton. We hypothesize that cytoskeletal organization and dynamics can provide important clues to the different malignant states of glioblastoma. In this study, we investigated cytoskeletal organization in glioblastoma cells with different subtype expression profiles, and cytoskeletal dynamics upon subtype transitions. Analysis of the morphological, migratory, and invasive properties of glioblastoma cells identified cytoskeletal components as phenotypic markers that can serve as diagnostic or prognostic tools. We also show that the cytoskeletal function and malignant properties of glioblastoma cells shift during subtype transitions induced by altered expression of the neurodevelopmental transcription factor SOX2. The potential of SOX2 re-expression to reverse the mesenchymal subtype into a more proneural subtype might open up strategies for novel glioblastoma treatments.

## Introduction

Glioblastoma is a highly invasive malignant tumor with poor patient prognosis, where the survival rate is about 15 months after diagnosis^1^. Patients are often treated by a combination of surgery, radiotherapy, and adjuvant chemotherapy. During the last decades, different treatment strategies have been investigated to improve patient quality of life and survival^2,3^. However, the increased survival to date is still not adequate, and one contributing factor appears to be the invasive nature of glioblastoma cells, which allows a cell fraction to remain after surgery, thereby leading to tumor reoccurrence.

Glioblastoma can be divided into at least three molecular subtypes based on their gene expression signatures and associated genetic perturbations: classical, proneural, and mesenchymal^4,5^. Several gene signatures and phenotypes have been linked to these subtypes, including loss of *NF1* for the mesenchymal subtype, *EGFR* amplification for the classical subtype, and *IDH1* mutations and high *SOX2* expression for the proneural subtype^4^. In cancer, during metastasis process, cells undergo metamorphosis known as epithelial-to-mesenchymal transition (EMT) and it has been shown that key regulators of actin dynamics are involved in EMT^6,7^.

In glioblastoma there is no epithelial counterpart but a transition from a non-mesenchymal to mesenchymal glioblastoma subtype have been reported to occur during tumor progression, for example, as describe previously, driven by loss of *NF1*^4^. Furthermore, EMT-related transcriptional regulators have been connected to the mesenchymal subtype including TWIST1^8^, SNAI1 and SNAI2 (SNAIL, SLUG)^9,10^, and TAZ/WWTR1^11^. The WNT pathway is also relevant in this context, and upon activation of this pathway, β-catenin is translocated to the nucleus. Through interactions with transcription cofactors such as TCF and LEF, β-catenin can serve as a transcription regulator and induce genes that drive EMT^12,13^. Although β-catenin is mainly regulated through the WNT signaling pathway, it can also be regulated by the Hippo or TGF-β pathways. β-Catenin is in addition a component of adherence junctions, which link the actin cytoskeleton to cell–cell adhesion complexes. From a morphological perspective, epithelial cells are characterized as differentiated and polarized, with well-developed cell–cell and cell–matrix junctions, and in general, epithelial cells have a low migratory index.

The cytoskeleton consists of actin filaments, intermediate filaments (IFs), and microtubules (MTs), and dynamic reorganization of the cytoskeleton is critical for the control of complex cellular behaviors, such as cell morphogenesis, contraction, and migration. In epithelial cells, actin filaments are associated with cell–cell junctions within the apical zone, and thereby, they form an adhesion belt^14–16^. In contrast, mesenchymal cells are more elongated and fibroblast-like, with prominent stress fibers. Glioblastoma is a highly invasive tumor and its invasive properties are likely to be under the control of the cytoskeletal dynamics. Therefore, a detailed study of the cytoskeletal organization and cell migration should provide important clues to the invasive behavior of glioblastoma.

It was recently described how glioblastoma cells can shift between proneural and mesenchymal subtypes under the influence of two regulatory elements, SOX2 and SFRP2. SOX2 is a transcription factor that regulates cancer stem cell properties, as it can bind and activate regulators of stem-like tumor-propagating cells that are involved in tumor progression and therapeutic resistance^17^. SOX2 also has roles in the regulation of proliferation and spheroid formation, which defines a subset of high-grade glioma cultures with lower responses to inhibitors of platelet-derived growth factor receptors and insulin-like growth factor receptors^18^. Moreover, *SOX2* down-regulation in glioblastoma mice models is associated with improved survival, inhibition of cell growth, and induction of cell death^19^. The WNT-signaling pathway has a role in cell proliferation, differentiation, adhesion, and migration^20^. Up-regulation of this pathway has also been described to be involved in radioresistance and chemoresistance^21^. In glioblastoma cells, overexpression of *SFRP2* (an upstream regulator of the WNT signaling pathway), inhibits tumor growth in vivo^22^. The *SFRP2* promoter is hypermethylated in around 50% of glioblastomas, which leads to loss of *SFRP2* expression^23^. This makes SOX2 and SFRP2 potential key elements in the regulation of glioblastoma subtypes.

In our previous study, we demonstrated that detailed knowledge of the organization and function of the cytoskeleton provides important clues to the malignancy of the highly aggressive malignant mesothelioma, a tumor that originates from the cell layer that covers the pleura and the peritoneum^24^. To investigate the relationships between cytoskeletal organization and glioblastoma subtypes here, 10 established glioblastoma cell lines were used. These cell lines belong to two established cell models for glioblastoma. The U-343 cell line originated from the same human glioblastoma biopsy from which U-343MG and U-343MGa cells were initially established^25^. U-343MGa Cl2:6 and U-343MGa 31L cells were subsequently derived from U-343MGa cells. The second group of cells used consists of six cell lines that are part of a panel of cells isolated from high-grade gliomas that were previously described by Hägerstrand et al.^26^.

The cell lines were analyzed for their cytoskeletal organization, and their migratory and invasive capacities. We also analyzed these parameters in highly invasive glioblastoma mesenchymal subtypes and less malignant glioblastoma proneural subtypes after modification of two key genetic elements: SOX2 and SFRP2 recently identified as subtype regulators. Based on our findings, we hypothesize that cytoskeletal organization and dynamics can provide important clues to the different malignant states of glioblastoma.

## Results

### Cytoskeletal organization of the glioblastoma cell lines

**Figure 1:**
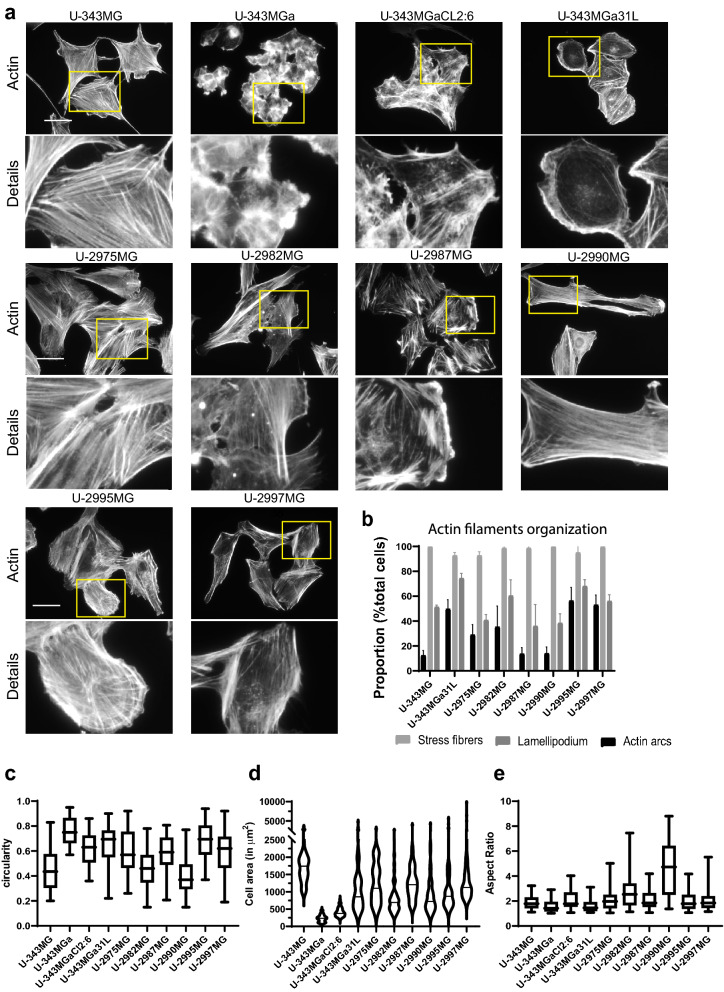
Actin filament organization and cell morphology. (**a**) Representative images of actin filament organization visualized by TRITC-conjugated phalloidin. Scale bar, 50 µm. (**b**) Analysis of the type of actin filament organization in each cell line. Quantification was performed from three independent experiments per cell line. Statistical significance was calculated with ANOVA-Tukey’s *post-hoc* tests and summarized in Supplementary Table S1. (**c–e**). Quantification of circularity (**c**), cell area (**d**), and aspect ratio (**e**) using ImageJ (n = 50 for each cell line). Statistical significance was calculated with ANOVA-Tukey’s *post-hoc* tests and summarized in Supplementary Table S2.

To study the organization of filamentous actin (F-actin), the cells were stained with fluorescently labeled phalloidin and analyzed by fluorescence microscopy. Stress fibers were visible in all of the cell lines, but they differed with regard to F-actin organization patterns and cell morphology (Fig. 1a,b). On this basis, the cells could morphologically be grouped into different categories: fibroblastic, intermediate, epithelioid. In addition, two cell lines (U-343MGa and U-343MGa CL2:6 grew in clusters, which made characterizations difficult because it was not always possible to identify individual cells.

Cells of cell lines U-2975MG and U-2987MG had a few lamellipodia, as 41% and 36% of the cells, respectively, and some had a few actin arcs, as 29% and 14% of the cells, respectively. In addition, in these cells the stress fiber bundles appeared shorter and thicker than for the other cell lines (Fig. 1a,b, Supplementary Tab. S1). These characteristics are indicative of an epithelioid phenotype. In contrast, cell lines U-2982MG and U-2990MG showed fibroblastic phenotypes according to their high aspect ratios (2.86, 4.65, respectively) and low circularity (0.46, 0.40, respectively). In addition, these cells had an abundance of stress fibers, which spanned the entire cell bodies (Fig. 1a,c,e, Supplementary Tab. S2). Cell lines U-343MGa 31L, U-2995MG, and U-2997MG showed phenotypes of an intermediate type, with relatively rounded cell shapes (circularity, 0.65, 0.69, 0.59, respectively) and intermediate areas (1.145, 1.157, 1.582 µm^2^, respectively), and with more actin arcs and lamellipodia (Fig. 1a,c,d, Supplementary Tab. S1). Cell line U-343MG had the largest cells (1.744 µm^2^), which were cuboid in shape (circularity, 0.46) and had fewer actin arcs, which were only seen for 12% of these cells (Fig. 1a–d, Supplementary Tab. S1). Finally, cell lines U-343MGa and U-343MGa Cl2:6 were highly circular (0.76, 0.62, respectively), small (area 236 and409 µm^2^, respectively), and grew in clusters (Fig. 1a,c,d, Supplementary Tab. S2).

**Figure 2:**
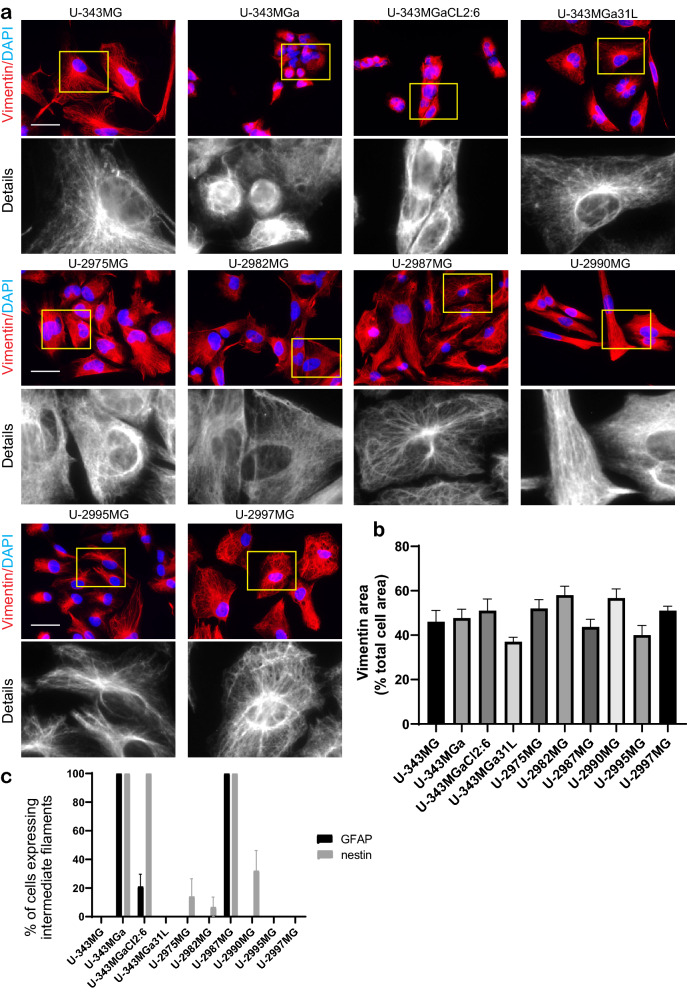
Intermediate filament organization and expression. (**a**) Representative images of vimentin organization, as stained with mouse anti-vimentin antibodies and AlexaFluor568-conjugated anti-mouse antibodies. Scale bar, 50 µm. (**b**) Quantification of the proportions (%) of the areas of the cell occupied by vimentin. Quantification was performed from three independent experiments using ImageJ. Statistical significance was calculated with ANOVA-Tukey’s *post-hoc* tests and summarized in Supplementary Table S3. (**c**) Quantification of the proportion (%) of cells expressing GFAP and nestin. Quantification was performed from three independent experiments per cell line. Statistical significance was calculated with ANOVA-Tukey’s *post-hoc* tests and summarized in Supplementary Table S4.

Vimentin is known to be expressed in mesenchymal cells and has been used as a diagnostic and prognostic marker in different cancers^27^. Vimentin was expressed in all glioblastoma cell lines analyzed in this study. However, the differences in the vimentin subcellular distributions classified the cells into two main groups. The first group comprised cell lines U-2987MG, U-2995MG, and U-343MGa 31L, where vimentin IFs occupied < 45% of the total cell area (Fig. 2b, Supplementary Tab. S3), and was localized mainly to the perinuclear area and on top of the nucleus (Fig. 2a). The second group comprised cell lines U-2982MG, U-2990MG, U-2997MG, and U-343MGa Cl2:6, where vimentin IFs spread over > 45% of the cell body (Fig. 2b, Supplementary Tab. S3), and the filaments were arranged in denser networks (Fig. 2a).

Two other IF proteins were also analyzed in these glioblastoma cell lines: glial fibrillary acidic protein (GFAP) and nestin. Cell lines U-2987MG and U-343MGa fully expressed both GFAP and nestin, whereas cell lines U-2995MG, U-2997MG, and U-343MG expressed neither GFAP nor nestin (Fig. 2c, Supplementary Fig. S1a, b). Few cells in cell lines U-2975MG, U-2982MG, and U-2990MG (14%, 7% and 32% of the cells respectively) expressed nestin but not GFAP, whereas for cell lines U-343MGa Cl2:6, 21% of the cells expressed GFAP, but did not express nestin (Fig. 2c, Supplementary Fig. S1a, b). Cell line U-343MGa 31L did not express nestin, however GFAP was express to some extent but not in a fibrillary form.

To visualize MT organization, the cells were stained with an antibody against α-tubulin. For cell lines U-2975MG, U-2990MG, U-2982MG, U-2995MG, U-343MG, and U-343MGa, the MTs spanned the entire body of the cells (Supplementary Fig. S2a), as a pattern of distribution that is often seen in cells with mesenchymal morphology. In contrast, for cell lines U-2987MG, U-2997MG, U-343MGa 31L, and U-343MGa Cl2:6, MTs appeared to be wrapped around the nucleus, and to have a more tangled organization in the rest of the cytoplasm; this is a MT organization that is often seen in cells with epithelial morphology (Supplementary Fig. S2a). Tubulin acetylation is indicative of MT stability, and acetylation of α-tubulin at lysine 40 is under the control of α-tubulin acetyltransferase 1 and histone deacetylase 6^28^. Cell lines U-343MG, U-2987MG, and U-2995MG showed the lowest ratios of acetylated MTs (0.20, 0.21, 0.32, respectively) and acetylated MTs were concentrated to the perinuclear area and absent from the cell edges (Supplementary Fig. S2a, b). Conversely, the ratios of acetylated MTs were the highest for cell lines U-2975MG, U-2990MG, U-2997MG, U-343MGa, and U-343MGa Cl2:6 (0.47 for cell lines U-2990MG, U-2997MG; 0.6 for U-343MGa cells) (Supplementary Figs. S2b, Supplementary Tab. S5). For these cells, acetylated MTs were visible in the perinuclear area and on MTs that were spread across the entire cytoplasm (Supplementary Figs. S2a). Cell lines U-343MGa 31L and U-2982MG had a ratio of acetylated MTs of around 0.4, meaning that MT acetylation was less prominent, but the pattern of distribution was like the previous cell lines (Supplementary Figs. S2a, b, Supplementary Tab. S5).

### Organization of cell–cell junctions in the glioblastoma cell lines

We next studied the organization of cell–cell junctions in the glioblastoma cell lines, using an antibody against β-catenin. The localization of β-catenin differed between the cell lines. For cell lines U-343MGa 31L and U-2987MG, β-catenin was located at the cell borders and created a well-defined framework at the cell–cell junctions (Supplementary Fig. S3a) and thin junction width (0.5 µm and 0.38 µm respectively) (Supplementary Fig. S3b), which is a phenotype frequently observed in cells with epithelial morphology. A similar organization was seen in cell lines U-343MGa and U-343MGa Cl2:6, with thin junction width (0.31 µm in each) but this was less distinguishable due to their clustered growth pattern (Supplementary Fig. S3a and b). In contrast, in cell lines U-343MG, U-2975MG, U-2982MG, U-2997MG, and U-2995MG, β-catenin was also localized to the cell borders, but the β-catenin organization appeared less defined and displayed broad junctions and a more serrated pattern (0.64 µm, 0.70 µm, 0.98 µm, 0.87 µm and 0.98 µm respectively) (Supplementary Fig. S3a). For cell lines U-2990MG the pattern of β-catenin localization was intermediate, with cells showing either a well-defined framework or serrated borders with width of 0.67 µm (Supplementary Fig. S3a).

### Migratory and invasive capacities of the glioblastoma cell lines

**Figure 3:**
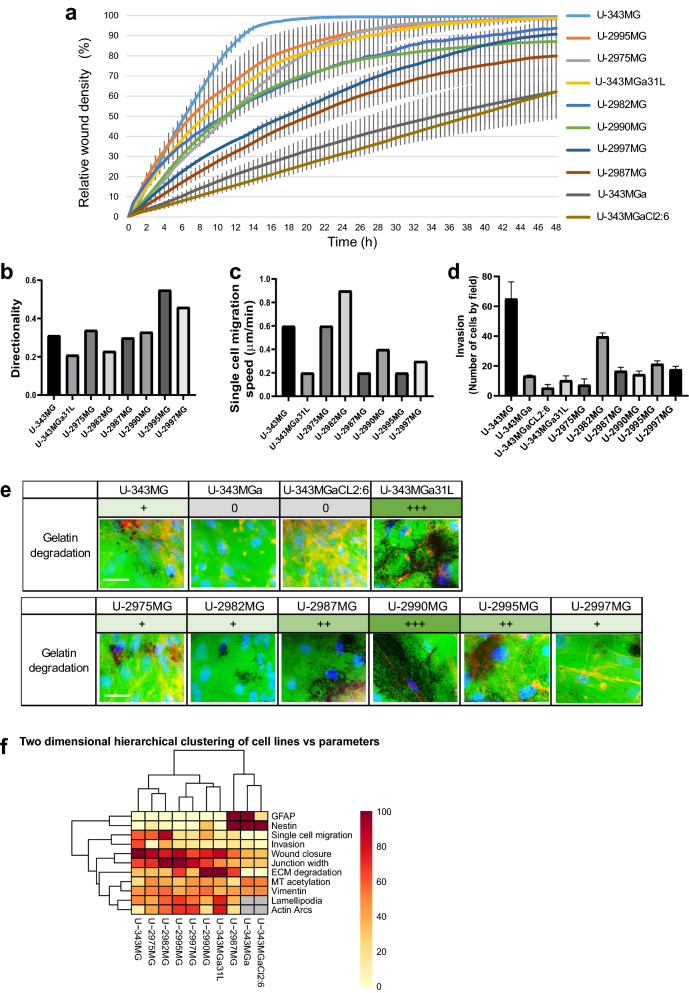
Migratory and invasion properties. (**a**) Quantification of wound closure over 48 h, using the IncuCyte imaging device. (**b,c**) Quantification of migration speed (**b**) and directionality of migration (**c**), performed for 25 cells per cell line using the Tracking Tool software. (**d**) Quantification of invasion, using cell migration through Transwell filters coated with Matrigel, and performed as three independent experiments, with statistical significance calculated with ANOVA-Tukey’s *post-hoc* tests and summarized in Supplementary Table S7. (**e**) Representative images of matrix degradation activity 48 h after seeding cells on coverslips coated with FITC-labeled gelatin, with estimated degradation activities given as 0 (no degradation) to +  +  + (high degradation). (**f**) Summary results of the phenotypes for the glioblastoma cell lines, shown as two-dimensional hierarchical clustering of cell lines *versus* parameters.

We next studied the migration and Matrigel invasion of the cell lines. To investigate the collective migration in two dimensions, wound closure was monitored for wounds inflicted in monolayers of cells, using the Incucyte imaging technique. Cell line U-343MG had the highest migratory capacity, with wound closure in < 24 h; for cell lines U-2975MG and U-2982MG, over the same period, these showed relative wound closures of 89% and 77%, respectively (Fig. 3a). The properties of this collective migration were reflected in the differences for single cell migration. Cell lines U-343MG, U-2975MG, and U-2982MG migrated with speeds of 0.6 μm/min, 0.6 μm/min and 0.9 μm/min, respectively (Fig. 3c), which agrees with the fibroblastic morphology. Cell line U-343MGa 31L was also relatively efficient for the collective cell migration, with relative wound closure of 87% after 24 h, although the single-cell velocity was low, at 0.15 μm/min (Fig. 3a,c). Cell line U-2990MG had a relative wound closure of 76% at 24 h, and migrated at 0.4 μm/min, whereas cell line U-2995MG showed relatively high wound closure, at 90% after 24 h, but slow single cell migration velocity, at 0.2 μm/min (Fig. 3a,c). Cell lines U-2987MG and U-2997MG were less motile, with relative wound closures of 57% and 63%, respectively, and single cell migration speeds of 0.2 μm/min and 0.3 μm/min, respectively (Fig. 3a,c). In contrast, cell lives U-343MGa and U-343MGa Cl2:6 had the lowest migratory capacity (Fig. 3a). In addition, even though cell lines U-343MG and U-2982MG migrated at high speeds, the directness of their migration was lower compared to cell lines U-2995MG and U-2997MG (Fig. 3b).

We next analyzed the invasive capacities of the glioblastoma cells using a Boyden type of chamber coated with Matrigel extracellular matrix components. Cell lines U-343MGa, U-2987MG, U-2990MG, U-2995MG, and U-2997MG had intermediate invasive capacities (14, 17, 14, 21, 18 cells per field, respectively), while cell lines U-343MGa CL2:6, U-343MGa 31L, and U-2975MG hardly invaded the Matrigel at all (5, 10, 7 cells per field, respectively) (Fig. 3d, Supplementary Tab. S7). Cell lines U-343MG and U-2982MG, which had the highest migratory index, also had the highest capacity for three-dimensional migration in the Matrigel invasion assay (Fig. 3d, Supplementary Tab. S7), where 40 and 65 cells per field, respectively, migrated through the membrane.

Another decisive factor for three-dimensional migration is the degradation of the extracellular matrix by cancer cells. To study this property, we analyzed the degradation of fluorescently labeled gelatin by these glioblastoma cell lines. Surprisingly, for some of the cell lines the gelatin degradation was ‘opposite’ to their Matrigel invasiveness. The most invasive were cell lines U-343MG and U-2982MG, and these showed low gelatin degradation, while cell lines U-343MGa 31L and U-2990MG were most efficient in the gelatin degradation, but they were not very invasive (Fig. 3d,e, Supplementary Tab. S7). Cell lines U-343MGa and U-343MGa CL2:6, which showed the least migration for all of these cell lines, were also the least invasive and the least efficient at gelatin degradation (Fig. 3a,d,e, Supplementary Tab. S7). Thus, the motility of these cells is controlled by different processes, and the collective migration efficiency does not always correlate with single speed migration, cell invasion, or gelatin degradation.

### SOX2 and SFRP2 have roles in regulation of subtype transition of the glioblastoma cell lines

As indicated above, based on their morphometric, migratory, and invasive properties, the glioblastoma cell lines were distributed along an axis, and they showed a predominant epithelioid morphology (cell lines U-343MGa 31L, U-343MGa, U-2975MG, U-2987MG, U-2995MG, U-2997MG) or a predominantly fibroblastic morphology (cell lines U-343MG, U-343MGa Cl2:6, U-2982MG, U-2990MG) (Fig. 3f). These data also indicated that cell lines U-2982MG and U-2987MG differed significantly from each other in terms of their migratory and invasive properties.

**Figure 4:**
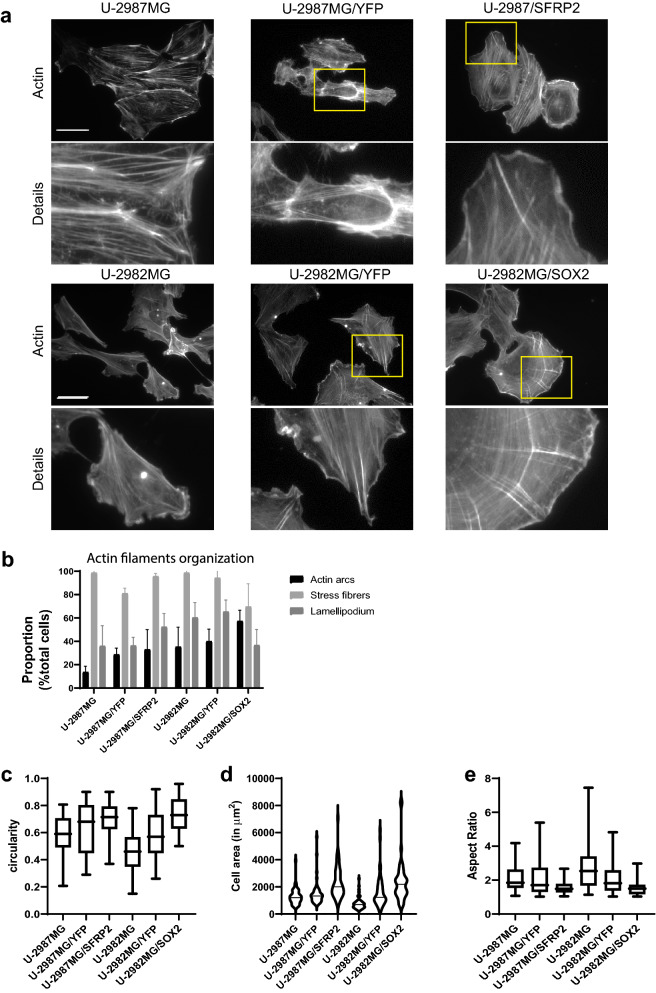
Actin filament organization and cell morphology in cell lines U-2982MG/SOX2 and U-2987MG/SFRP2. (**a**) Representative images of actin filament organization, visualized using TRITC-conjugated phalloidin. Scale bar, 50 µm. (**b**) Analysis of the type of actin organization in each cell line. Quantification was performed from three independent experiment in at least 150 cells per cell line. Statistical significance was calculated with ANOVA-Tukey’s *post-hoc* tests and summarized in Supplementary Table S8. (**c–e**) Quantification of circularity (**c**), cell area (**d**), and aspect ratio (**e**), using ImageJ (n = 50 for each cell line). Statistical significance was calculated with ANOVA-Tukey’s post-hoc tests and summarized in Supplementary Table S9.

**Figure 5:**
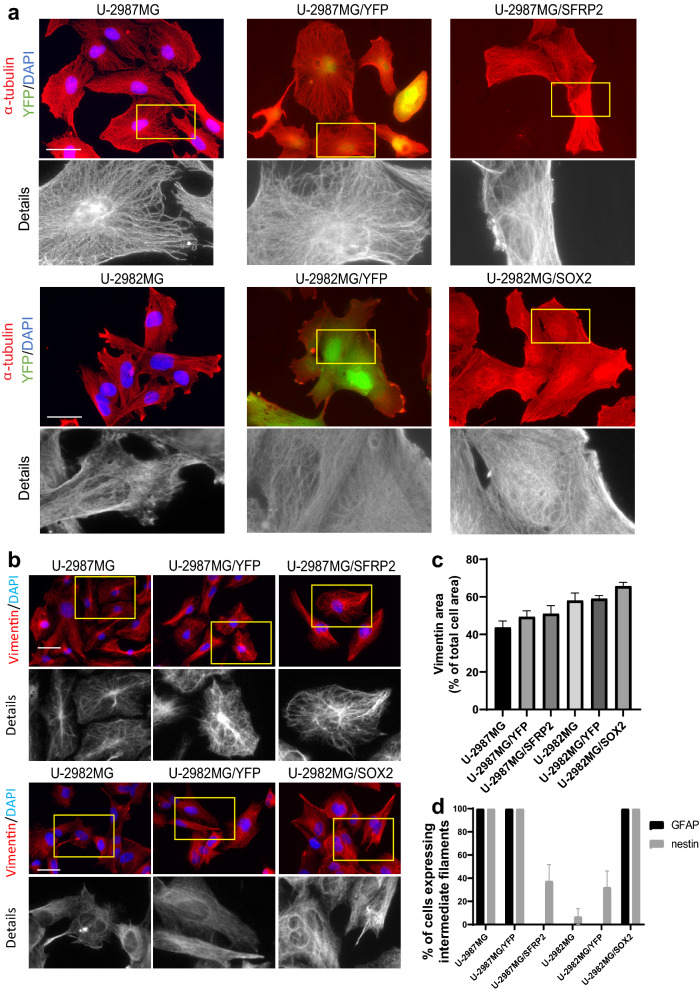
α-tubulin and vimentin organization in cell lines U-2982MG/SOX2 and U-2987MG/SFRP2. (**a**) Representative images of α-tubulin organization, as stained with rabbit anti–α-tubulin antibodies and AMCA-conjugated donkey anti-rabbit antibodies. (**b**) Representative images of vimentin organization, as stained with mouse anti-vimentin antibodies and AlexaFluor568-conjugated goat anti-mouse antibodies. Scale bars, 50 µm (A, B). (**c**) Quantification of the proportion (%) of the area of the cell occupied by vimentin. Quantification was performed from three independent experiments using ImageJ, and statistical significance was calculated with ANOVA-Tukey’s *post-hoc* tests and summarized in Supplementary Table S10. (**d**) Quantification of the proportion (%) of cells expressing GFAP and nestin. Quantification was performed from three independent experiments per cell line. Statistical significance was calculated with ANOVA-Tukey’s *post-hoc* tests and summarized in Supplementary Table S10.

The cell lines also differed significantly in their cytoskeleton organization. Previous studies have shown that based on its gene expression signature, cell line U-2982MG can be stratified as a mesenchymal subtype glioblastoma^19^. In contrast, cell line U-2987MG shows a proneural signature, because of high expression of the transcription factors *SOX2* and *PROX1*^19,29^. Transcriptome analysis by RNAseq or gene arrays reported previously indicated that two separate groups of glioblastoma cell lines can be distinguished^19,25^. For genes related to mesenchymal properties, including migration, invasion, and chemoresistance, cell lines U-2975MG and U-2982MG show greater expression than cell lines U-2987MG, U-2990MG, U-2995MG, and U-2997MG. Examples of such genes are *GREM1*, *FN1*, *LUM*, *SPARC*, and *COL1A1*^30–34^. The transcription factor SOX2 has been shown to regulate glioblastoma gene expression, to shift the cells from proneural subtype to mesenchymal subtype, and SOX2 can be suppressed by SFRP2^29^. To understand more about the critical factors that are decisive for the two major glioblastoma subtypes, we used two cell lines that were produced in an earlier study: cell line U-2982MG with stable overexpression of *SOX2* (U-2982MG/SOX2), and cell line U-2987MG with stable overexpression of *SFRP2* (U-2987MG/SFRP2). To study the dynamics of cytoskeletal structure during glioblastoma subtype transition, the cell line model used included cell lines U-2982MG, U-2982MG/YFP, U-2982MG/SOX2, U-2987MG, U-2987MG/YFP, and U-2987MG/SFRP2, which thus represented two cell types with mesenchymal subtype gene expression signature (i.e. U-2982MG and U-2987MG/SFRP2), two cell types with proneural subtype gene expression signature (i.e., U-2982MG/SOX2, and U-2987MG), and two transfection control cell lines (i.e., U-2982MG/YFP and U-2987MG/YFP) (Fig. 3f)^29^. Cell line U-2982MG/SOX2 had more cells with actin arcs and fewer lamellipodia (57%, 37% of the cells, respectively), as compared to cell line U-2982MG (35%, 61%, respectively) (Fig. 4a–e, Supplementary Tab. S8). In contrast, cell line U-2987MG/SFRP2 had gained a fibroblast-like, mesenchymal appearance, with long stress fibers and with lamellipodia in 52% of the cells, as compared to cell line U-2987MG with 36% (Fig. 4a–e, Supplementary Tab. S8). Cell line U-2982MG/SOX2 showed a more tangled MT network compared to control cell line U-2982MG, whereas for cell line U-2987MG/SFRP2, the MT network appeared straight and was less wrapped around the nucleus compared to control cell line U-2987MG/YFP (Fig. 5a).

We then analyzed the expression and localization of the IF proteins nestin, GFAP, and vimentin in these cell lines. Cell line U-2987MG/SFRP2 did not express GFAP anymore, contrary to control cell line U-2987MG/YFP, while cell line U-2982MG/SOX2 fully expressed GFAP contrary to cell line U-2982MG and U-2982MG/YFP (Fig. 5d; Supplementary Fig. S4, Supplementary Tab. S10). Compared to the control cell lines, there was lower nestin expression for cell line U-2987MG/SFRP2 (37% of cells), and higher nestin expression for U-2982MG/SOX2 cells (100% of cells) (Fig. 5d; Supplementary Fig. S4b, Supplementary Tab. S10). The SOX2 and SFRP2 expression in cell lines U-2982MG and U-2987MG, respectively, did not significantly impact vimentin expression in terms of its area or its distribution, in agreement with the transcriptomic data (Fig. 5b,c, Supplementary Tab. S10)^29^.

### SOX2 and SFRP2 have roles in regulation of cell–cell junction integrity in the glioblastoma cell lines

Cell–cell junctions were visualized with an antibody against β-catenin. For control U-2987MG/YFP cells, β-catenin localized to well-defined cell–cell junctions and at the cell borders with an average thickness size of 0.5 µm (Supplementary Fig. S5a and b). For control cell line U-2982MG/YFP, the cell–cell junctions appeared serrated with an average thickness of 0.85 µm (Supplementary Fig. S5a and b). The localization of β-catenin in cell line U-2987MG/SFRP2 was more similar to control cell line U-2982MG/YFP and the junctions were thicker (0.58 µm), while β-catenin localization in cell line U-2982MG/SOX2 was more similar to control cell line U-2987MG/YFP and the junctions were thinner (0.67 µm) (Supplementary Fig. S5a).

### SOX2 and SFRP2 expression regulate the migratory properties of the glioblastoma cell lines

**Figure 6:**
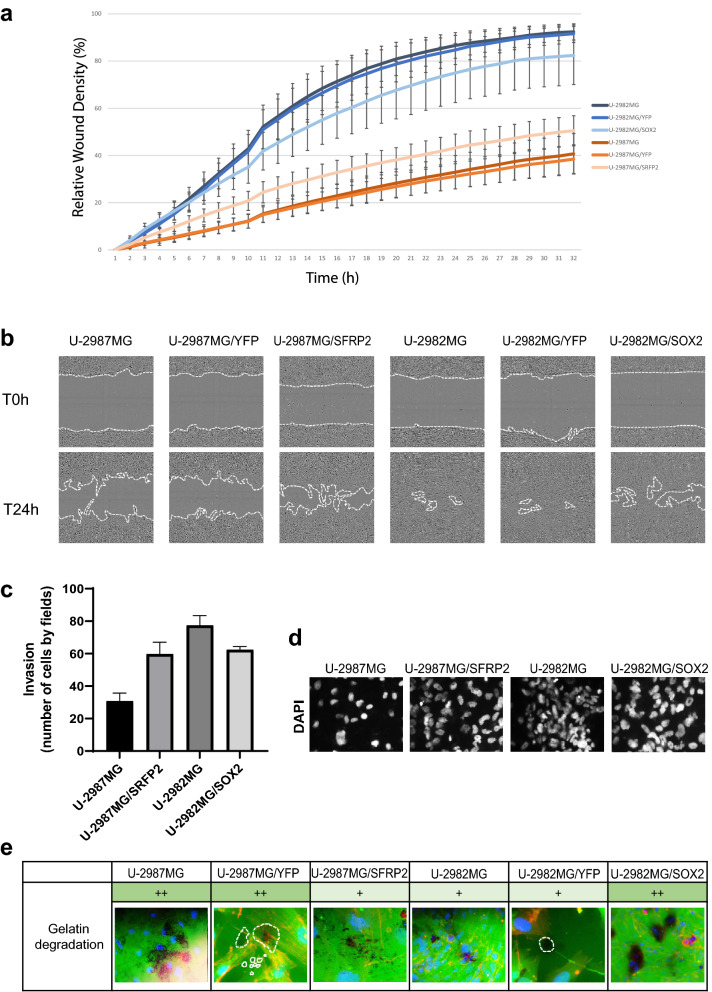
Migratory and invasive properties of cell lines U-2982MG/SOX2 and U-2987MG/SFRP2. (**a**) Quantification of wound closure over 48 h, using the IncuCyte imaging device to track live-cell motility. (**b**) Representative images of wound closure in the cell lines, at t = 0 h and after 24 h. (**c**) Quantification of invasion using cell migration through Transwell filters coated with Collagen 1. Statistical significance was calculated with ANOVA-Tukey’s *post-hoc* tests and summarized in Supplementary Table S12. (**d**) Representative images of invasive cells on the lower face of the membrane, as stained with DAPI. (**e**) Representative images of matrix degradation activity 48 h after seeding the cells on coverslips coated with FITC-labeled gelatin, with degradation activity from 0 (no degradation) to +  +  + (high degradation).

Cell line U-2982MG was more efficient in the single cell and collective migration and invasion assays (Fig. 3a,c,d, Supplementary Tab. S7), whereas cell line U-2987MG showed greater gelatin degradation (Fig. 3e). Cell line U-2982MG/SOX2 showed lower relative wound closure (77% vs. 88% for cell line U-2982MG/YFP after 24 h) (Fig. 6a,b). Cell line U-2982MG/SOX2 invasion was also less pronounce compared to cell line U-2982MG (62 vs. 77 invasive cells per field, respectively) (Fig. 6c,d, Supplementary Tab. S12); however, gelatin degradation was higher for cell line U-2982MG/SOX2 (Fig. 6e). SFRP2 overexpression in cell line U-2987MG increase collective migration (relative wound density of 44% after 24 h Vs 34% for U-2987MG/YFP) and cell invasiveness (60 vs. 30 invasive cells per field for U-2987MG/YFP) through Collagen 1 (Fig. 6a–e, Supplementary Tab. S12) but reduced gelatin degradation.

In summary, SFRP2 expression in cell line U-2987MG switched key characteristics of the epithelioid cell line into a fibroblastic phenotype. In contrast, SOX2 expression in cell line U-2982MG resulted in conversion of the fibroblastic phenotype into an epithelioid phenotype (Supplementary Fig. S6).

## Discussion

The overall aim of the current study was to determine whether information on cytoskeletal organization and dynamics could provide clues about differences between glioblastoma subtypes. In a previous study on malignant mesothelioma, we reported consistent differences in the organization of F-actin and intermediate filaments that reflected the aggressiveness of the cells from the malignant mesotheliomas of the patients^24^. In the glioblastoma cell lines here, the F-actin organization differed visibly between the cell lines that were of a more epithelioid subtype compared to those of a more mesenchymal subtype. Thus, cell lines U-343MGa 31L, U-2987MG, and U-2995MG were more circular and had shorter stress fibers, and cell lines U-343MG, U-2975MG, U-2982MG, and U-2990MG were more elongated and had long stress fibers that spanned the entire cell body. This information allowed us to group the cell lines according to an epithelial morphology or a mesenchymal morphology. Cell line U-2987MG is positioned toward a predominantly epithelioid morphology, cell line U-2982MG toward a predominantly fibroblastic morphology, and the other cell lines fit into intermediate positions according to the two-dimensional hierarchical clustering (Fig. 3f).

Epithelial-to-mesenchymal transition is characterized by different morphological alterations, which include loss of cell–cell junctions. These intercellular attachment points are made up of different proteins, including cadherins and β-catenin, and they are linked to actin filaments. We observed clear differences in β-catenin organization between the different cell lines here. The clearest differences were seen between cell lines U-2982MG and U-2987MG, and between cell lines U-343MG and U-343MGa 31L. Cell lines U-343MGa 31L and U-2987MG had strong accumulation of β-catenin at the borders between adjacent cells, which is indicative of an epithelioid morphology. In contrast, cell lines U-343MG and U-2982MG had broad and serrated β-catenin-positive areas, which indicates mesenchymal cell characteristics. We did not detect any expression of β-catenin in the nuclei. In summary, β-catenin localization is a clear indicator of epithelioid *versus* fibroblastic glioblastoma morphology.

A previous analysis of malignant mesothelioma cells demonstrated correlation between migratory potential and tubulin acetylation^24^. Cells with a high migratory index generally have a lower ratio of acetylated tubulin. This is in line with the prevailing concept that acetylated MTs are more stable than non-acetylated MTs, and migrating cells have been shown to have less stable MTs^28,35^. Surprisingly, we did not find such a correlation in glioblastoma cells. Despite clear differences in cell migration, we did not find any major differences in the ratios of acetylated MTs, with the exception of cell line U-343MG, which was highly motile, and cell line U-2987MG, which showed low collective and single-cell migration. Thus based on these results, tubulin acetylation is not an ideal prognostic marker for glioblastoma migratory and invasive potential.

One important observation from this study is that there were significant differences in the migratory properties of the different glioblastoma cell lines. Mesenchymal cell lines U-343MG and U-2982MG had high migratory abilities in the single-cell migration, wound closure, and Matrigel invasion assays. In contrast, cell lines U-343MGa 31L and, in particular, U-2987MG had much lower migratory potentials in all of these assays. Interestingly, the cell migration did not correlate with the matrix degrading activities; indeed, this appeared to be largely the opposite, whereby the least migratory cells showed the most efficient matrix degradation. We also reported this concept in our studies of malignant mesothelioma^24^. The gelatin degradation assay is mainly dependent on invadopodia formation and activity. In contrast, the Matrigel used in the invasion assays is composed of collagen I, collagen IV, and laminin, which together with fibronectin, proteoglycans and hyaluronic acid, constitute the main components of the glioblastoma extracellular matrix environment^36^. This suggests that these two categories of glioblastoma use different modes to spread, and in a cancer setting, they need different treatments to counteract their migratory potential and invasive potential.

We have shown that glioblastoma cells of epithelioid and mesenchymal phenotypes have clear differences in their cytoskeletal fingerprints, which makes it possible to stratify them into subtypes. Cell line U-2987MG had obvious epithelioid properties, and was a representative of this subtype, whereas cell line U-2982MG had typical mesenchymal characteristics, and was a representative of this subtype. Cell line U-2987MG had a low migratory index, which was seen as a low migratory speed for single-cell migration, and for wound closure and Matrigel invasion. In contrast, cell line U-2982MG showed efficient wound closure and invasion, and these cells migrated with the highest speed of all cell lines tested. Importantly, we identified that the two established regulatory elements SOX2 and SFRP2 have key roles in the subtype cytoskeleton specification. During differentiation from a proneural to a mesenchymal subtype, SOX2 expression is lost. Interestingly, re-expression of SOX2 in the mesenchymal type cell line U-2982MG resulted in regression of the phenotype into an epithelioid cell type, based on the cytoskeletal characteristics and migratory behavior. Expression of SFRP2 in cell line U-2987MG resulted in transition to a mesenchymal phenotype with a more specific cytoskeletal organization increasing collective migration and invasion, as SFRP2 has been shown to act as a SOX2 antagonist in glioblastoma cells^29^. In conclusion, elucidating different mechanisms in migration and invasion as well as the potential of SOX2 re-expression to reverse the mesenchymal subtype into a more proneural subtype, should thus open a strategy to alternative treatments in glioblastoma. Moreover, as it has been promising in other type of cancer, further investigations in cytoskeletal reorganization in glioblastoma during tumor progression could be interesting to use as diagnostic tool.

## Methods

### Cell culture

All cell line were taken from lab Monika Nistér, Karolinska Institutet, Stockholm, Sweden (and from her former supervisor Bengt Westermark, Uppsala University, Uppsala, Sweden). Briefly, the U-343MG and U-343MGa cell lines were established from the same human glioblastoma multiform biopsy. U-343MGa Cl2:6 and U-343MGa 31L cells were derived from the U-343MGa cells^25,37,38^. Cell lines U-2975MG, U-2987MG, U-2995MG, and U-2997MG were diagnosed as grade 3–4 according to the World Health Organization, and cell lines U-2982MG and U-2990MG were diagnosed as grade 4^26^. Cell lines U-2982MG/YFP, U-2982MG/SOX2, U-2987MG/YFP, and U-2987MG/SFRP2 were establish by transduction of SOX2 or SFRP2 overexpression lentivirus vectors into cell lines U-2982MG and U-2987MG, respectively^29^. YFP was introduced into both cell lines U-2982MG and U-2987MG in the same way, as controls. All of the cells were cultured in Dulbecco’s modified Eagle’s medium supplemented with 10% decomplemented fetal bovine serum, and penicillin/streptomycin (1000 U/mL and 1000 μg/mL, respectively). The cell lines were maintained at 37 °C in a humidified incubator with an atmosphere of 5% CO_2_.

### Antibodies

The following commercial antibodies and reagents were used: mouse monoclonal anti-α-tubulin antibodies (T9026); mouse monoclonal anti-vimentin antibodies (V6630); mouse monoclonal anti-acetylated α-tubulin antibodies (T6793); tetramethyl rhodamine isothiocyanate (TRITC)-conjugated phalloidin (P1951); 4′,6-diamidino-2-phenylindole dihydrochloride (DAPI; D9542) (all Sigma-Aldrich, St. Louis, MO, USA); mouse monoclonal anti-nestin antibodies (#33,475; Cell Signaling, Danvers, MA, USA); mouse monoclonal anti-β-catenin antibodies (#610,153; BD Biosciences, Franklin Lakes, NJ, USA); rabbit polyclonal anti-α-tubulin antibodies (ab18251; Abcam, Cambridge, UK); rabbit polyclonal anti-GFAP antibodies (Z0334) (Agilent Technologies, Santa Clara, CA, USA); AlexaFluor 488-conjugated donkey anti-mouse antibodies (A21202); AlexaFluor 488-conjugated goat anti-rabbit antibodies (A11008); AlexaFluor568-conjugated donkey anti-rabbit antibodies (A10042) (all Thermo Fisher Scientific, Waltham, MA, USA); AMCA conjugated donkey anti-rabbit antibodies (AB-2340602; Jackson Immuno Research Europe, Cambridge, UK).

### Immunofluorescence

Cells were seeded on glass coverslips and incubated for 24 h. The cells were fixed in 3% paraformaldehyde in phosphate-buffered saline (PBS) for 25 min at 37 °C, and washed three times with PBS. The cells were permeabilized with 0.2% Triton X-100 in PBS for 5 min, washed three times with PBS, and then incubated in 5% fetal bovine serum in PBS (FBS/PBS) for 30 min at room temperature. After further PBS washes, the cells were incubated in 5% FBS/PBS with the primary antibody for 1 h, washed 3 times with PBS, and incubated in 5% FBS/PBS with the secondary antibody, DAPI, and TRITC-phalloidin. The cover slips were mounted on slides with mounting medium (Southern Biotechnology Associates, Birmingham, AL, USA), and photographed using a fluorescence microscope (AxioVert 40 CFL; Carl Zeiss AG, Oberkochen, Germany) equipped with a camera (AxioCam; Carl Zeiss AG) and powered by the AxioVision software (Carl Zeiss AG).

### Matrigel invasion

Invasion assays were carried out as described elsewhere^24^. For cell lines U-2982MG, U-2982MG/YFP, U-2982MG/SOX2, U-2987MG, U-2987MG/YFP and U-2987MG/SFRP2 cells were seeded on invasion chambers coated with Collagen 1. The cells were then photographed under the microscope (as described for immunofluorescence), and the numbers of cells per field were counted.

### Migration assay

Wound closure assays were performed using an imaging system (IncuCyte Zoom; Essen Bioscience, Ann Arbor, MI, USA), as described elsewhere^24^. For cell lines U-2982MG, U-2982MG/YFP, U-2982MG/SOX2, U-2987MG, U-2987MG/YFP and U-2987MG/SFRP2 cells were seeded on 96 wells plate coated with Collagen 1, and recorded for 32 h every hour. Single cell migration was recorded over a period of 20 h using a microscope (AxioObserver.Z1; Carl Zeiss AG) equipped with a camera (AxioCam MRm; Carl Zeiss AG). Images were obtained every 5 min over 20 h. The single-cell trajectories were quantified using the Image Tracking Pro v2.1 software (Gradientech AB, Uppsala, Sweden).

### Gelatin degradation assay

The degradation analysis of the extracellular matrix was carried out with Gelatin Invadopodia assay kits (Merck-Millipore, Darmstadt, Germany), as described elsewhere^24^.

## Supplementary Information

Supplementary Information.
