# Supplementary material for: Altered cytoskeletal status in the transition from proneural to mesenchymal glioblastoma subtypes
Source: Sci Rep. 2022 Jun 14;12:9838. doi: 10.1038/s41598-022-14063-7 (PMC9197936; doi:10.1038/s41598-022-14063-7)
Supplement: Supplementary file 1 — Supplementary Information. [file 41598_2022_14063_MOESM1_ESM.pdf]

Supplementary Figure S1

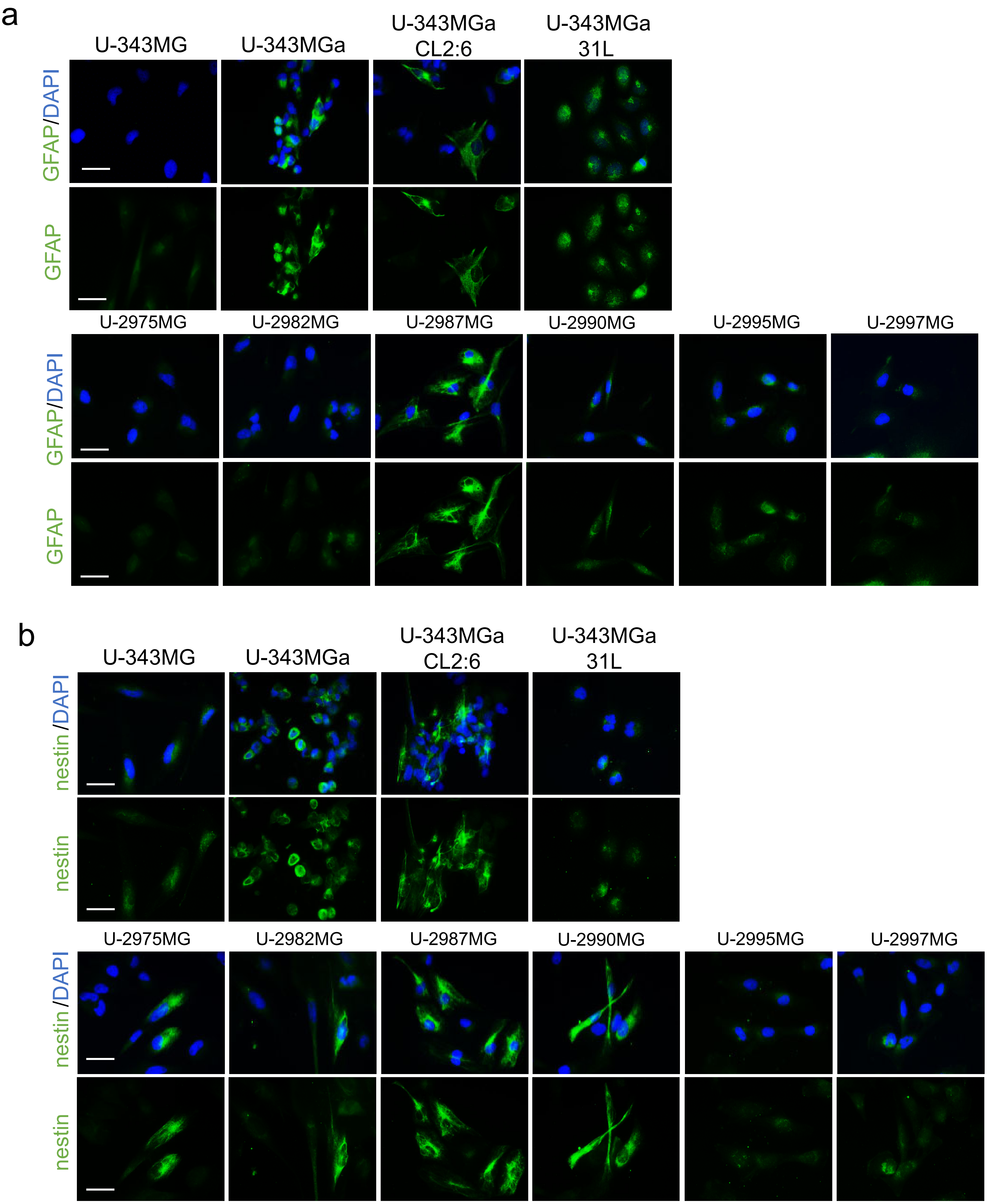

Supplementary Figure S1. Visualization of GFAP and nestin intermediate filaments.
a. Representative images of GFAP intermediate filaments, as visualized with mouse anti-GFAP antibodies followed by AlexaFluor488-conjugated anti-mouse antibodies. b. Representative images of nestin intermediate filaments visualized with mouse anti-nestin antibodies followed by AlexaFluor488-conjugated anti-mouse antibodies. Scale bars, 50 µm.

Supplementary Figure S2

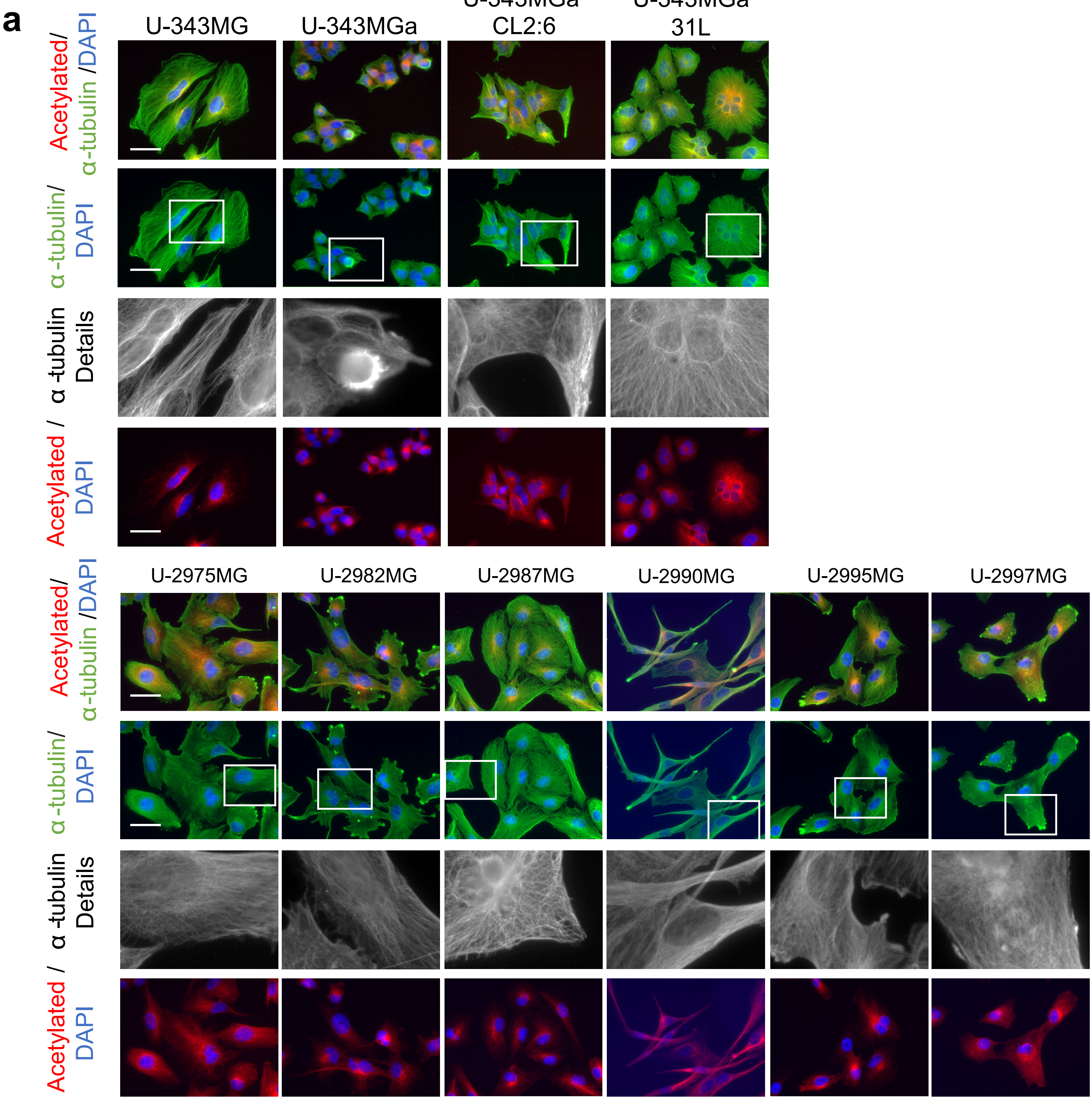

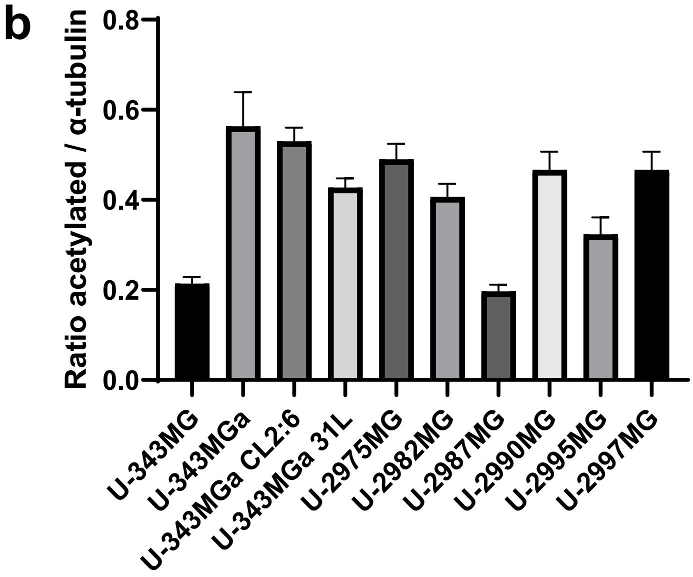

Supplementary Figure S2. Visualization of dynamic and stable microtubules.
a. Representative images of microtubule organization in the U-343MG–derived cell lines, as visualized with rabbit antibodies against α-tubulin followed by AlexaFluor488-conjugated donkey anti-rabbit antibodies. Stable microtubules were visualized with mouse antibodies against acetylated tubulin followed by AlexaFluor568-conjugated goat anti-mouse antibodies. Scale bars, 50 µm. b. Ratio of area occupied by acetylated α-tubulin to area occupied by α-tubulin. Quantification was performed from three independent experiments for each cell line, and analyzed using ImageJ. Statistical significance was calculated with one-way ANOVA with Tukey's post-hoc tests and summarized in Supplementary Table S5.

Supplementary Figure S3

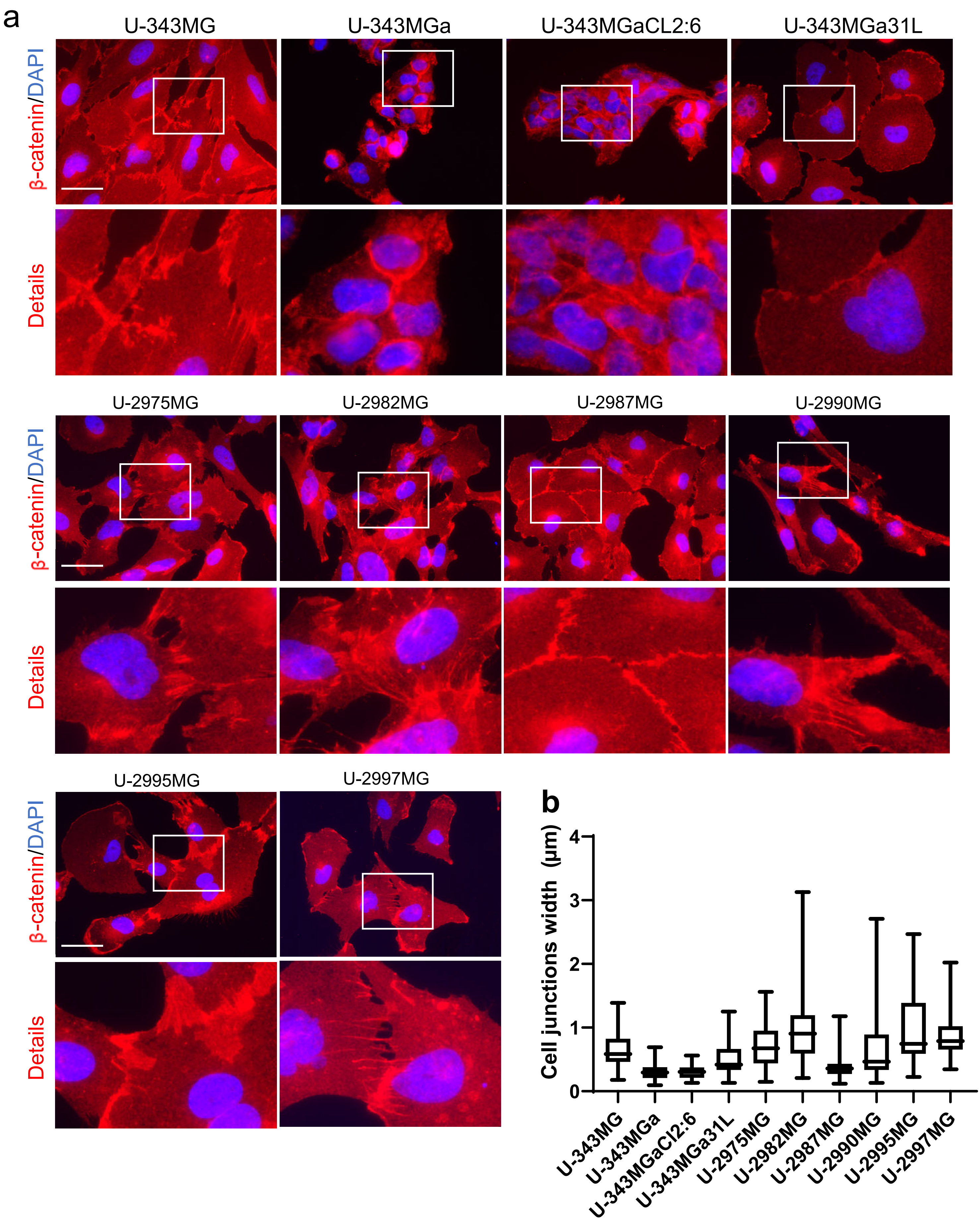

Supplementary Figure S3. Subcellular localization of β-catenin.
a. Representative images of β-catenin localization, as visualized with mouse anti-β-catenin antibodies followed by AlexaFluor568-conjugated goat anti-mouse antibodies. Scale bars, 50 μm. b. Quantification of the width of β-catenin in the areas of the cells. Quantification was performed using ImageJ, as performed from three independent experiments, and statistical significance was calculated with ANOVA-Tukey's post-hoc tests and summarized in Supplementary Table S6.

Supplementary Figure S4

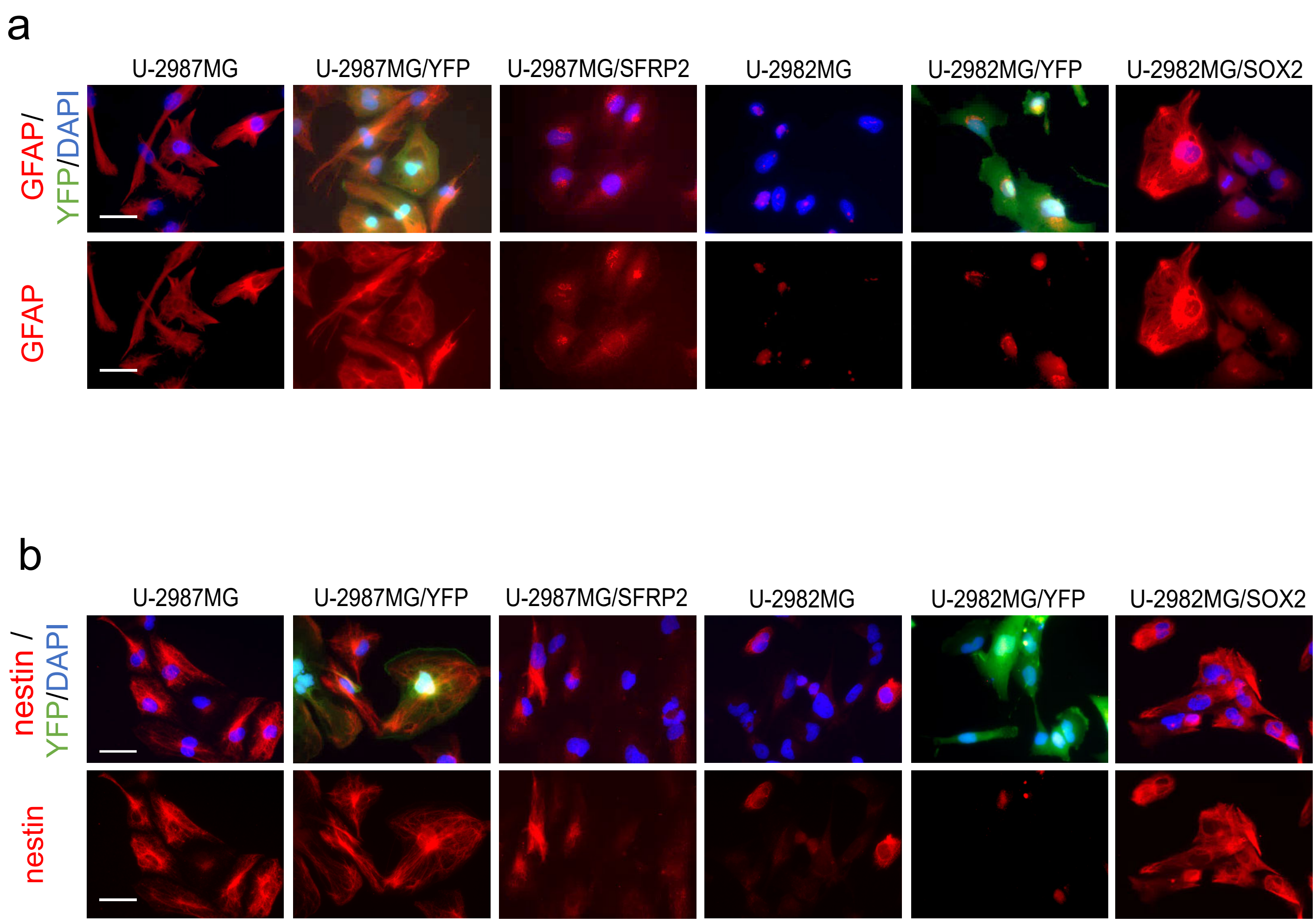

Supplementary Figure S4. Visualization of GFAP and nestin intermediate filaments in cell lines U-2982MG/SOX2 and U-2987MG/SFRP2.
a. Representative images of GFAP intermediate filaments, as visualized with mouse anti-GFAP antibodies followed by AlexaFluor568-conjugated anti-mouse antibodies. b. Representative images of nestin intermediate filaments, as visualized with mouse anti-nestin antibodies followed by AlexaFluor568-conjugated anti-mouse antibodies. Scale bars, 50 μm.

Supplementary Figure S5

a

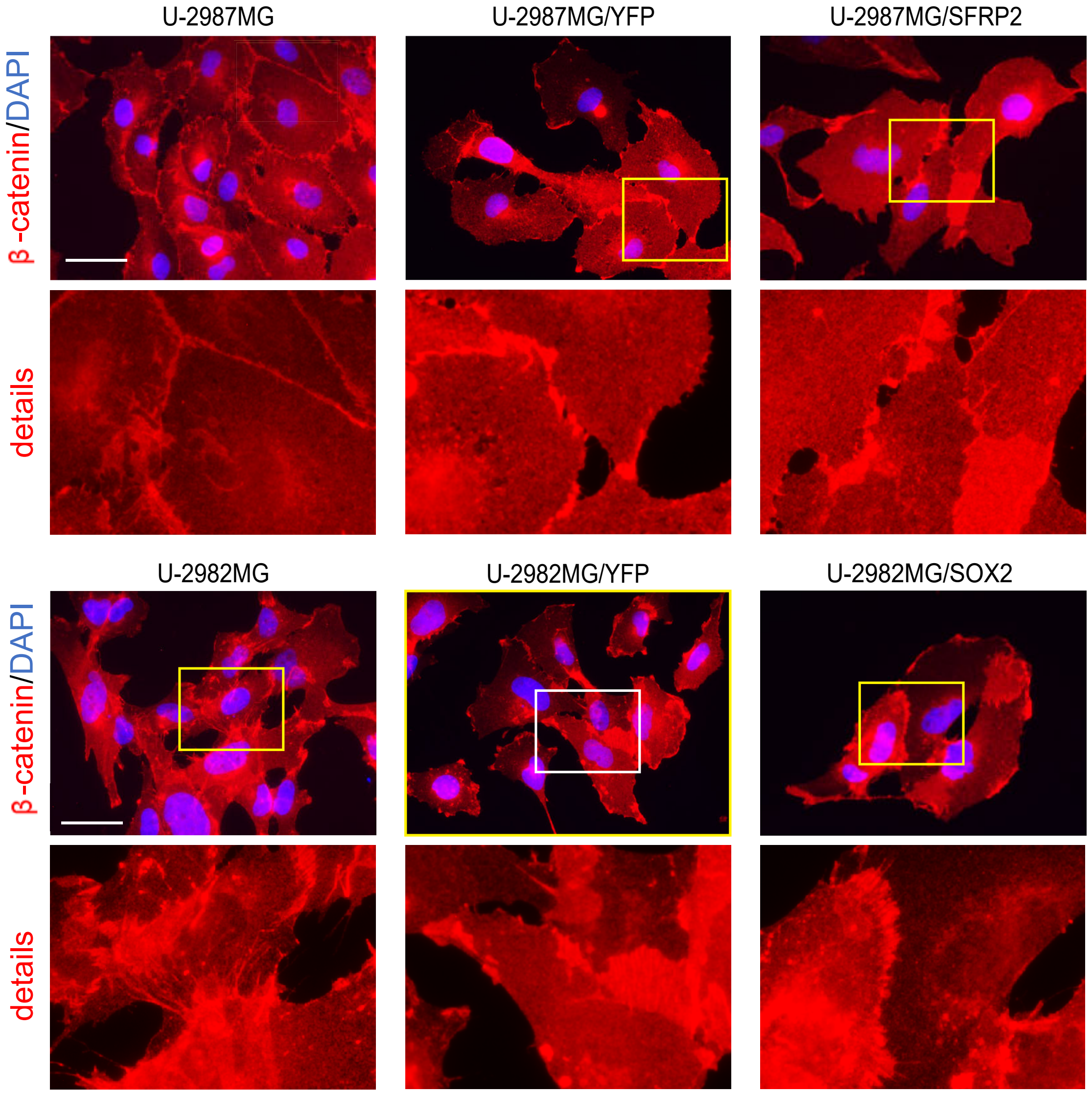

b

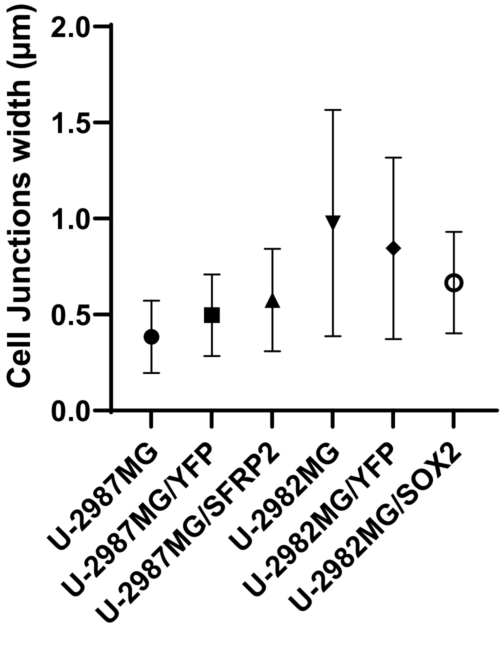

Supplementary Figure S5. Visualization of β-catenin in cell lines U-2982MG/SOX2 and U-2987MG/SFRP2. a. Representative images of β-catenin localization, as stained with mouse anti-β-catenin antibodies and AlexaFluor568-conjugated goat anti-mouse antibodies. Scale bar, 50 µm. b. Quantification of the width of β-catenin on the areas of the cell. Quantification was performed using ImageJ, as performed from three independent experiments, and statistical significance was calculated with ANOVA-Tukey's post-hoc tests and summarized in Supplementary Table S11.

Supplementary Figure S6

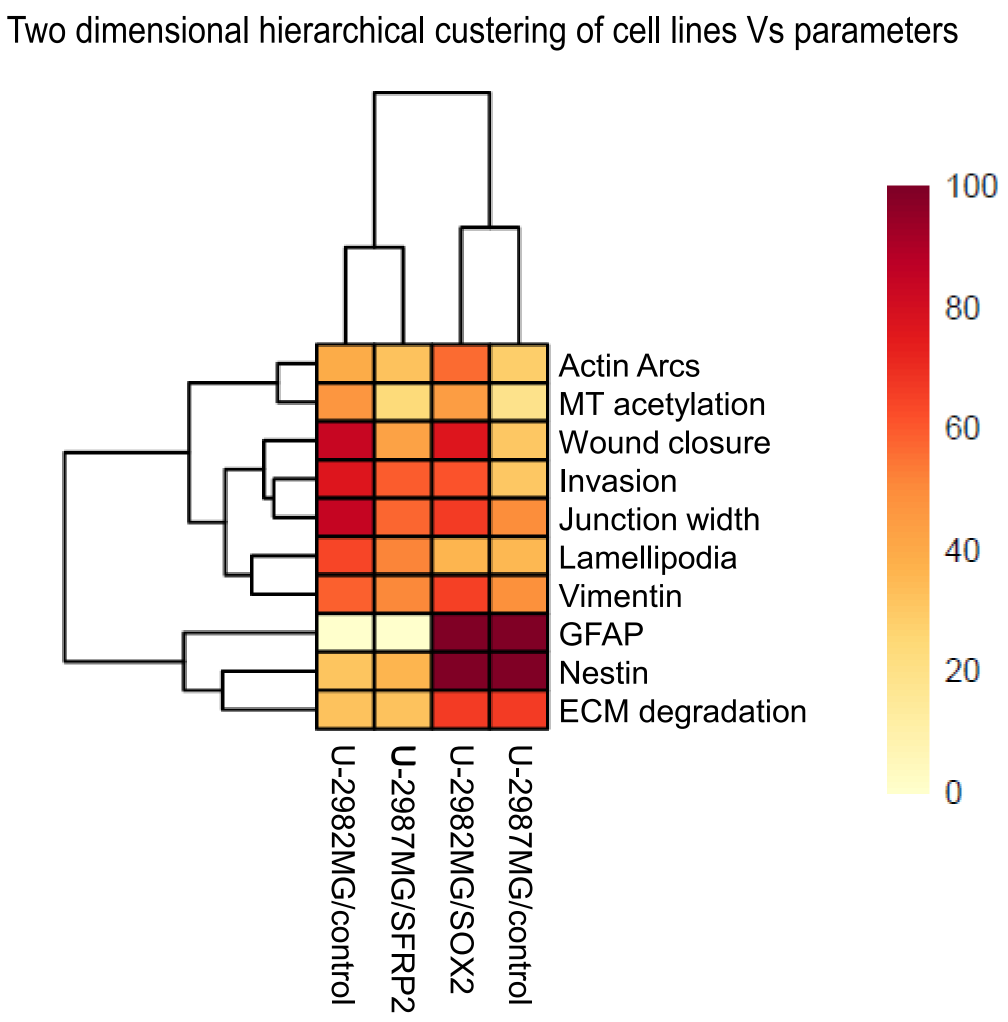

Supplementary Figure S6. Summary of phenotypes in cell lines U-2982MG/SOX2 and U-2987MG/SFRP2.
Summary results of the phenotypes for cell lines U-2982MG (control), U-2982MG/SOX2, U-2987MG (control), and U-2987MG/SFRP2, with two-dimensional hierarchical clustering of cell lines versus parameters.

Supplementary Table S1

Complete list of the significances of the data presented in Figure 1b, calculated by two-way ANOVA with Tukey's *post-hoc* tests.

	Actin arc		Stress fibrers		Lamellipodia	
Tukey's multiple comparisons test	Summary	Adjusted P Value	Summary	Adjusted P Value	Summary	Adjusted P Value
U-343MG vs. U-343MGa31L	****	<0,0001	ns	0,9263	**	0,0095
U-343MG vs. U-2975MG	ns	0,1356	ns	0,9263	ns	0,6531
U-343MG vs. U-2982MG	**	0,0095	ns	>0,9999	ns	0,8142
U-343MG vs. U-2987MG	ns	>0,9999	ns	>0,9999	ns	0,2116
U-343MG vs. U-2990MG	ns	>0,9999	ns	>0,9999	ns	0,4069
U-343MG vs. U-2995MG	****	<0,0001	ns	0,991	ns	0,1356
U-343MG vs. U-2997MG	****	<0,0001	ns	>0,9999	ns	0,994
U-343MGa31L vs. U-2975MG	*	0,0276	ns	>0,9999	****	<0,0001
U-343MGa31L vs. U-2982MG	ns	0,2859	ns	0,9743	ns	0,314
U-343MGa31L vs. U-2987MG	****	<0,0001	ns	0,9743	****	<0,0001
U-343MGa31L vs. U-2990MG	****	<0,0001	ns	0,9263	****	<0,0001
U-343MGa31L vs. U-2995MG	ns	0,9546	ns	>0,9999	ns	0,9654
U-343MGa31L vs. U-2997MG	ns	0,9993	ns	0,9416	ns	0,0729
U-2975MG vs. U-2982MG	ns	0,9654	ns	0,9743	*	0,0424
U-2975MG vs. U-2987MG	ns	0,2116	ns	0,9743	ns	0,994
U-2975MG vs. U-2990MG	ns	0,2347	ns	0,9263	ns	>0,9999
U-2975MG vs. U-2995MG	**	0,0011	ns	>0,9999	**	0,0011
U-2975MG vs. U-2997MG	**	0,0059	ns	0,9416	ns	0,2116
U-2982MG vs. U-2987MG	*	0,0177	ns	>0,9999	**	0,005
U-2982MG vs. U-2990MG	*	0,0205	ns	>0,9999	*	0,0152
U-2982MG vs. U-2995MG	*	0,0238	ns	0,9987	ns	0,9087
U-2982MG vs. U-2997MG	ns	0,0942	ns	>0,9999	ns	0,9962
U-2987MG vs. U-2990MG	ns	>0,9999	ns	>0,9999	ns	>0,9999
U-2987MG vs. U-2995MG	****	<0,0001	ns	0,9987	****	<0,0001
U-2987MG vs. U-2997MG	****	<0,0001	ns	>0,9999	*	0,0369
U-2990MG vs. U-2995MG	****	<0,0001	ns	0,991	***	0,0003
U-2990MG vs. U-2997MG	****	<0,0001	ns	>0,9999	ns	0,0942
U-2995MG vs. U-2997MG	ns	0,9993	ns	0,994	ns	0,5101

Supplementary Table S2

Complete list of the significances of the data presented in Figure 1c-e, calculated by one-way ANOVA with Tukey's *post-hoc* tests.

	Circularity		Area		Aspect ratio	
Tukey's multiple comparisons test	Significance	Adjusted p value	Significance	Adjusted p value	Significance	Adjusted p value
U-343MG vs. U-343MGa	****	<0,0001	****	<0,0001	ns	0,9273
U-343MG vs. U-343MGaCl2:6	****	<0,0001	****	<0,0001	ns	0,9423
U-343MG vs. U-343MGa31L	****	<0,0001	**	0,003	ns	0,9597
U-343MG vs. U-2975MG	****	<0,0001	*	0,0353	ns	0,8723
U-343MG vs. U-2982MG	ns	>0,9999	****	<0,0001	****	<0,0001
U-343MG vs. U-2987MG	**	0,001	ns	0,058	ns	0,975
U-343MG vs. U-2990MG	ns	0,8009	****	<0,0001	****	<0,0001
U-343MG vs. U-2995MG	****	<0,0001	**	0,004	ns	0,9995
U-343MG vs. U-2997MG	***	0,0004	ns	0,9864	ns	0,9903
U-343MGa vs. U-343MGaCl2:6	***	0,0002	ns	0,9777	ns	0,159
U-343MGa vs. U-343MGa31L	**	0,0076	****	<0,0001	ns	>0,9999
U-343MGa vs. U-2975MG	****	<0,0001	****	<0,0001	ns	0,096
U-343MGa vs. U-2982MG	****	<0,0001	**	0,0098	****	<0,0001
U-343MGa vs. U-2987MG	****	<0,0001	****	<0,0001	ns	0,2299
U-343MGa vs. U-2990MG	****	<0,0001	***	0,0003	****	<0,0001
U-343MGa vs. U-2995MG	ns	0,4653	****	<0,0001	ns	0,5267
U-343MGa vs. U-2997MG	****	<0,0001	****	<0,0001	ns	0,3106
U-343MGaCl2:6 vs. U-343MGa31L	ns	0,9968	****	<0,0001	ns	0,2128
U-343MGaCl2:6 vs. U-2975MG	ns	>0,9999	****	<0,0001	ns	>0,9999
U-343MGaCl2:6 vs. U-2982MG	****	<0,0001	ns	0,262	*	0,0189
U-343MGaCl2:6 vs. U-2987MG	ns	0,9828	****	<0,0001	ns	>0,9999
U-343MGaCl2:6 vs. U-2990MG	****	<0,0001	*	0,0245	****	<0,0001
U-343MGaCl2:6 vs. U-2995MG	ns	0,3009	****	<0,0001	ns	0,9998
U-343MGaCl2:6 vs. U-2997MG	ns	0,996	****	<0,0001	ns	>0,9999
U-343MGa31L vs. U-2975MG	ns	0,9418	ns	0,9995	ns	0,1334
U-343MGa31L vs. U-2982MG	****	<0,0001	ns	0,3301	****	<0,0001
U-343MGa31L vs. U-2987MG	ns	0,5959	ns	0,9973	ns	0,298
U-343MGa31L vs. U-2990MG	****	<0,0001	ns	0,8899	****	<0,0001
U-343MGa31L vs. U-2995MG	ns	0,8673	ns	>0,9999	ns	0,618
U-343MGa31L vs. U-2997MG	ns	0,734	ns	0,104	ns	0,3903
U-2975MG vs. U-2982MG	***	0,0001	ns	0,0629	*	0,0361
U-2975MG vs. U-2987MG	ns	0,9997	ns	>0,9999	ns	>0,9999
U-2975MG vs. U-2990MG	****	<0,0001	ns	0,4527	****	<0,0001
U-2975MG vs. U-2995MG	ns	0,1065	ns	0,9998	ns	0,9978
U-2975MG vs. U-2997MG	ns	>0,9999	ns	0,4494	ns	>0,9999
U-2982MG vs. U-2987MG	**	0,0019	*	0,0385	*	0,0107
U-2982MG vs. U-2990MG	ns	0,7085	ns	0,9967	****	<0,0001
U-2982MG vs. U-2995MG	****	<0,0001	ns	0,2849	**	0,0017
U-2982MG vs. U-2997MG	***	0,0008	****	<0,0001	**	0,0062
U-2987MG vs. U-2990MG	****	<0,0001	ns	0,3443	****	<0,0001
U-2987MG vs. U-2995MG	*	0,0146	ns	0,9987	ns	>0,9999
U-2987MG vs. U-2997MG	ns	>0,9999	ns	0,5658	ns	>0,9999
U-2990MG vs. U-2995MG	****	<0,0001	ns	0,856	****	<0,0001
U-2990MG vs. U-2997MG	****	<0,0001	***	0,0005	****	<0,0001
U-2995MG vs. U-2997MG	*	0,0286	ns	0,1269	ns	>0,9999

Supplementary Table S3

Complete list of the significances of the data presented in Figure 2b, calculated by one-way ANOVA with Tukey's *post-hoc* tests.

	Vimentin area	
Tukey's multiple comparisons test	Summary	Adjusted P Value
U-343MG vs. U-343MGa	ns	>0,9999
U-343MG vs. U-343MGaCl2:6	ns	0,8643
U-343MG vs. U-343MGa31L	ns	0,2171
U-343MG vs. U-2975MG	ns	0,7066
U-343MG vs. U-2982MG	*	0,0381
U-343MG vs. U-2987MG	ns	0,9991
U-343MG vs. U-2990MG	ns	0,0863
U-343MG vs. U-2995MG	ns	0,7066
U-343MG vs. U-2997MG	ns	0,8643
U-343MGa vs. U-343MGaCl2:6	ns	0,9872
U-343MGa vs. U-343MGa31L	ns	0,0863
U-343MGa vs. U-2975MG	ns	0,9353
U-343MGa vs. U-2982MG	ns	0,1048
U-343MGa vs. U-2987MG	ns	0,9592
U-343MGa vs. U-2990MG	ns	0,2171
U-343MGa vs. U-2995MG	ns	0,4032
U-343MGa vs. U-2997MG	ns	0,9872
U-343MGaCl2:6 vs. U-343MGa31L	*	0,0104
U-343MGaCl2:6 vs. U-2975MG	ns	>0,9999
U-343MGaCl2:6 vs. U-2982MG	ns	0,521
U-343MGaCl2:6 vs. U-2987MG	ns	0,4606
U-343MGaCl2:6 vs. U-2990MG	ns	0,7645
U-343MGaCl2:6 vs. U-2995MG	ns	0,0707
U-343MGaCl2:6 vs. U-2997MG	ns	>0,9999
U-343MGa31L vs. U-2975MG	**	0,0053
U-343MGa31L vs. U-2982MG	***	0,0001
U-343MGa31L vs. U-2987MG	ns	0,5831
U-343MGa31L vs. U-2990MG	***	0,0002
U-343MGa31L vs. U-2995MG	ns	0,9938
U-343MGa31L vs. U-2997MG	*	0,0104
U-2975MG vs. U-2982MG	ns	0,7066
U-2975MG vs. U-2987MG	ns	0,3007
U-2975MG vs. U-2990MG	ns	0,9037
U-2975MG vs. U-2995MG	*	0,0381
U-2975MG vs. U-2997MG	ns	>0,9999
U-2982MG vs. U-2987MG	**	0,0083
U-2982MG vs. U-2990MG	ns	>0,9999
U-2982MG vs. U-2995MG	***	0,0007
U-2982MG vs. U-2997MG	ns	0,521
U-2987MG vs. U-2990MG	*	0,02
U-2987MG vs. U-2995MG	ns	0,9761
U-2987MG vs. U-2997MG	ns	0,4606
U-2990MG vs. U-2995MG	**	0,0017
U-2990MG vs. U-2997MG	ns	0,7645
U-2995MG vs. U-2997MG	ns	0,0707

Supplementary Table S4

Complete list of the significances of the data presented in Figure 2c, calculated by one-way ANOVA with Tukey's *post-hoc* tests.

	GFAP		nestin	
Tukey's multiple comparisons test	Summary	Adjusted P Value	Summary	Adjusted P Value
U-343MG vs. U-343MGa	****	<0,0001	****	<0,0001
U-343MG vs. U-343MGaCl2:6	***	0,0002	****	<0,0001
U-343MG vs. U-343MGa31L	ns	>0,9999	ns	>0,9999
U-343MG vs. U-2975MG	ns	>0,9999	*	0,0349
U-343MG vs. U-2982MG	ns	>0,9999	ns	0,8089
U-343MG vs. U-2987MG	****	<0,0001	****	<0,0001
U-343MG vs. U-2990MG	ns	>0,9999	****	<0,0001
U-343MG vs. U-2995MG	ns	>0,9999	ns	>0,9999
U-343MG vs. U-2997MG	ns	>0,9999	ns	>0,9999
U-343MGa vs. U-343MGaCl2:6	****	<0,0001	ns	>0,9999
U-343MGa vs. U-343MGa31L	****	<0,0001	****	<0,0001
U-343MGa vs. U-2975MG	****	<0,0001	****	<0,0001
U-343MGa vs. U-2982MG	****	<0,0001	****	<0,0001
U-343MGa vs. U-2987MG	ns	>0,9999	ns	>0,9999
U-343MGa vs. U-2990MG	****	<0,0001	****	<0,0001
U-343MGa vs. U-2995MG	****	<0,0001	****	<0,0001
U-343MGa vs. U-2997MG	****	<0,0001	****	<0,0001
U-343MGaCl2:6 vs. U-343MGa31L	***	0,0002	****	<0,0001
U-343MGaCl2:6 vs. U-2975MG	***	0,0002	****	<0,0001
U-343MGaCl2:6 vs. U-2982MG	***	0,0002	****	<0,0001
U-343MGaCl2:6 vs. U-2987MG	****	<0,0001	ns	>0,9999
U-343MGaCl2:6 vs. U-2990MG	***	0,0002	****	<0,0001
U-343MGaCl2:6 vs. U-2995MG	***	0,0002	****	<0,0001
U-343MGaCl2:6 vs. U-2997MG	***	0,0002	****	<0,0001
U-343MGa31L vs. U-2975MG	ns	>0,9999	*	0,0349
U-343MGa31L vs. U-2982MG	ns	>0,9999	ns	0,8089
U-343MGa31L vs. U-2987MG	****	<0,0001	****	<0,0001
U-343MGa31L vs. U-2990MG	ns	>0,9999	****	<0,0001
U-343MGa31L vs. U-2995MG	ns	>0,9999	ns	>0,9999
U-343MGa31L vs. U-2997MG	ns	>0,9999	ns	>0,9999
U-2975MG vs. U-2982MG	ns	>0,9999	ns	0,7136
U-2975MG vs. U-2987MG	****	<0,0001	****	<0,0001
U-2975MG vs. U-2990MG	ns	>0,9999	**	0,0022
U-2975MG vs. U-2995MG	ns	>0,9999	*	0,0349
U-2975MG vs. U-2997MG	ns	>0,9999	*	0,0349

Supplementary Table S4 (following table)

Complete list of the significances of the data presented in Figure 2c, calculated by one-way ANOVA with Tukey's *post-hoc* tests.

	GFAP		nestin	
Tukey's multiple comparisons test	Summary	Adjusted P Value	Summary	Adjusted P Value
U-2975MG vs. U-2982MG	ns	>0,9999	ns	0,7136
U-2975MG vs. U-2987MG	****	<0,0001	****	<0,0001
U-2975MG vs. U-2990MG	ns	>0,9999	**	0,0022
U-2975MG vs. U-2995MG	ns	>0,9999	*	0,0349
U-2975MG vs. U-2997MG	ns	>0,9999	*	0,0349
U-2982MG vs. U-2987MG	****	<0,0001	****	<0,0001
U-2982MG vs. U-2990MG	ns	>0,9999	****	<0,0001
U-2982MG vs. U-2995MG	ns	>0,9999	ns	0,8089
U-2982MG vs. U-2997MG	ns	>0,9999	ns	0,8089
U-2987MG vs. U-2990MG	****	<0,0001	****	<0,0001
U-2987MG vs. U-2995MG	****	<0,0001	****	<0,0001
U-2987MG vs. U-2997MG	****	<0,0001	****	<0,0001
U-2990MG vs. U-2995MG	ns	>0,9999	****	<0,0001
U-2990MG vs. U-2997MG	ns	>0,9999	****	<0,0001
U-2995MG vs. U-2997MG	ns	>0,9999	ns	>0,9999

Supplementary Table S5

Complete list of the significances of the data presented in Figure supplementary 2b, calculated by one-way ANOVA with Tukey’s *post-hoc* tests.

	Ratio acetylated / α-tubulin	
Tukey's multiple comparisons test	Summary	Adjusted P Value
U-343MG vs. U-343MGa	****	<0,0001
U-343MG vs. U-343MGaCl2:6	****	<0,0001
U-343MG vs. U-343MGa31L	****	<0,0001
U-343MG vs. U-2975MG	****	<0,0001
U-343MG vs. U-2982MG	***	0,0001
U-343MG vs. U-2987MG	ns	0,9999
U-343MG vs. U-2990MG	****	<0,0001
U-343MG vs. U-2995MG	*	0,0475
U-343MG vs. U-2997MG	****	<0,0001
U-343MGa vs. U-343MGaCl2:6	ns	0,9813
U-343MGa vs. U-343MGa31L	**	0,0076
U-343MGa vs. U-2975MG	ns	0,3868
U-343MGa vs. U-2982MG	**	0,0018
U-343MGa vs. U-2987MG	****	<0,0001
U-343MGa vs. U-2990MG	ns	0,1108
U-343MGa vs. U-2995MG	****	<0,0001
U-343MGa vs. U-2997MG	ns	0,1108
U-343MGaCl2:6 vs. U-343MGa31L	ns	0,0731
U-343MGaCl2:6 vs. U-2975MG	ns	0,943
U-343MGaCl2:6 vs. U-2982MG	*	0,0193
U-343MGaCl2:6 vs. U-2987MG	****	<0,0001
U-343MGaCl2:6 vs. U-2990MG	ns	0,5755
U-343MGaCl2:6 vs. U-2995MG	****	<0,0001
U-343MGaCl2:6 vs. U-2997MG	ns	0,5755
U-343MGa31L vs. U-2975MG	ns	0,5755
U-343MGa31L vs. U-2982MG	ns	0,9996
U-343MGa31L vs. U-2987MG	****	<0,0001
U-343MGa31L vs. U-2990MG	ns	0,943
U-343MGa31L vs. U-2995MG	ns	0,0731
U-343MGa31L vs. U-2997MG	ns	0,943
U-2975MG vs. U-2982MG	ns	0,2372
U-2975MG vs. U-2987MG	****	<0,0001
U-2975MG vs. U-2990MG	ns	0,9985
U-2975MG vs. U-2995MG	***	0,0009
U-2975MG vs. U-2997MG	ns	0,9985
U-2982MG vs. U-2987MG	****	<0,0001
U-2982MG vs. U-2990MG	ns	0,6417
U-2982MG vs. U-2995MG	ns	0,2372
U-2982MG vs. U-2997MG	ns	0,6417
U-2987MG vs. U-2990MG	****	<0,0001
U-2987MG vs. U-2995MG	*	0,0153
U-2987MG vs. U-2997MG	****	<0,0001
U-2990MG vs. U-2995MG	**	0,0047
U-2990MG vs. U-2997MG	ns	>0,9999
U-2995MG vs. U-2997MG	**	0,0047

Supplementary Table S6

Complete list of the significances of the data presented in Figure supplementary 3b, calculated by one-way ANOVA with Tukey's *post-hoc* tests.

	Junctions width	
Tukey's multiple comparisons test	Summary	Adjusted P Value
U-343MG vs. U-343MGa	***	0,0003
U-343MG vs. U-343MGaCl2:6	***	0,0004
U-343MG vs. U-343MGa31L	ns	0,6393
U-343MG vs. U-2975MG	ns	0,9985
U-343MG vs. U-2982MG	***	0,0004
U-343MG vs. U-2987MG	*	0,017
U-343MG vs. U-2990MG	ns	>0,9999
U-343MG vs. U-2995MG	***	0,0002
U-343MG vs. U-2997MG	ns	0,0727
U-343MGa vs. U-343MGaCl2:6	ns	>0,9999
U-343MGa vs. U-343MGa31L	ns	0,2075
U-343MGa vs. U-2975MG	****	<0,0001
U-343MGa vs. U-2982MG	****	<0,0001
U-343MGa vs. U-2987MG	ns	0,9886
U-343MGa vs. U-2990MG	****	<0,0001
U-343MGa vs. U-2995MG	****	<0,0001
U-343MGa vs. U-2997MG	****	<0,0001
U-343MGaCl2:6 vs. U-343MGa31L	ns	0,2448
U-343MGaCl2:6 vs. U-2975MG	****	<0,0001
U-343MGaCl2:6 vs. U-2982MG	****	<0,0001
U-343MGaCl2:6 vs. U-2987MG	ns	0,9935
U-343MGaCl2:6 vs. U-2990MG	****	<0,0001
U-343MGaCl2:6 vs. U-2995MG	****	<0,0001
U-343MGaCl2:6 vs. U-2997MG	****	<0,0001
U-343MGa31L vs. U-2975MG	ns	0,1568
U-343MGa31L vs. U-2982MG	****	<0,0001
U-343MGa31L vs. U-2987MG	ns	0,8601
U-343MGa31L vs. U-2990MG	ns	0,3601
U-343MGa31L vs. U-2995MG	****	<0,0001
U-343MGa31L vs. U-2997MG	****	<0,0001
U-2975MG vs. U-2982MG	**	0,0099
U-2975MG vs. U-2987MG	***	0,0008
U-2975MG vs. U-2990MG	ns	>0,9999
U-2975MG vs. U-2995MG	**	0,0065
U-2975MG vs. U-2997MG	ns	0,4302
U-2982MG vs. U-2987MG	****	<0,0001
U-2982MG vs. U-2990MG	**	0,0022
U-2982MG vs. U-2995MG	ns	>0,9999
U-2982MG vs. U-2997MG	ns	0,9165
U-2987MG vs. U-2990MG	**	0,0039
U-2987MG vs. U-2995MG	****	<0,0001
U-2987MG vs. U-2997MG	****	<0,0001
U-2990MG vs. U-2995MG	**	0,0013
U-2990MG vs. U-2997MG	ns	0,2004
U-2995MG vs. U-2997MG	ns	0,8721

Supplementary Table S7

Complete list of the significances of the data presented in Figure 3d, calculated by one-way ANOVA with Tukey's *post-hoc* tests.

	Invasion	
Tukey's multiple comparisons test	Summary	Adjusted P Value
U-343MG vs. U-343MGa	****	<0,0001
U-343MG vs. U-343MGaCl2:6	****	<0,0001
U-343MG vs. U-343MGa31L	****	<0,0001
U-343MG vs. U-2975MG	****	<0,0001
U-343MG vs. U-2982MG	****	<0,0001
U-343MG vs. U-2987MG	****	<0,0001
U-343MG vs. U-2990MG	****	<0,0001
U-343MG vs. U-2995MG	****	<0,0001
U-343MG vs. U-2997MG	****	<0,0001
U-343MGa vs. U-343MGaCl2:6	ns	0,4489
U-343MGa vs. U-343MGa31L	ns	0,9965
U-343MGa vs. U-2975MG	ns	0,7837
U-343MGa vs. U-2982MG	****	<0,0001
U-343MGa vs. U-2987MG	ns	0,9925
U-343MGa vs. U-2990MG	ns	>0,9999
U-343MGa vs. U-2995MG	ns	0,4489
U-343MGa vs. U-2997MG	ns	0,9587
U-343MGaCl2:6 vs. U-343MGa31L	ns	0,9081
U-343MGaCl2:6 vs. U-2975MG	ns	0,9999
U-343MGaCl2:6 vs. U-2982MG	****	<0,0001
U-343MGaCl2:6 vs. U-2987MG	ns	0,0961
U-343MGaCl2:6 vs. U-2990MG	ns	0,3019
U-343MGaCl2:6 vs. U-2995MG	**	0,0061
U-343MGaCl2:6 vs. U-2997MG	ns	0,0551
U-343MGa31L vs. U-2975MG	ns	0,9965
U-343MGa31L vs. U-2982MG	****	<0,0001
U-343MGa31L vs. U-2987MG	ns	0,7317
U-343MGa31L vs. U-2990MG	ns	0,9747
U-343MGa31L vs. U-2995MG	ns	0,1148
U-343MGa31L vs. U-2997MG	ns	0,5613
U-2975MG vs. U-2982MG	****	<0,0001
U-2975MG vs. U-2987MG	ns	0,2607
U-2975MG vs. U-2990MG	ns	0,6191
U-2975MG vs. U-2995MG	*	0,0207
U-2975MG vs. U-2997MG	ns	0,1619
U-2982MG vs. U-2987MG	****	<0,0001
U-2982MG vs. U-2990MG	****	<0,0001
U-2982MG vs. U-2995MG	**	0,0014
U-2982MG vs. U-2997MG	***	0,0002
U-2987MG vs. U-2990MG	ns	0,9995
U-2987MG vs. U-2995MG	ns	0,9367
U-2987MG vs. U-2997MG	ns	>0,9999
U-2990MG vs. U-2995MG	ns	0,6191
U-2990MG vs. U-2997MG	ns	0,9925
U-2995MG vs. U-2997MG	ns	0,9856

Supplementary Table S8

Complete list of the significances of the data presented in Figure 4b, calculated by one-way ANOVA with Tukey's *post-hoc* tests.

	Actin arc		Stress fibrers		Lamellipodia	
Tukey's multiple comparisons test	Summary	Adjusted P Value	Summary	Adjusted P Value	Summary	Adjusted P Value
U-2987MG vs. U-2987MG/YFP	ns	0,5688	ns	0,39	ns	>0,9999
U-2987MG vs. U-2987MG/SFRP2	ns	0,2936	ns	0,9994	ns	0,4769
U-2987MG vs. U-2982MG	ns	0,1863	ns	>0,9999	ns	0,1032
U-2987MG vs. U-2982MG/YFP	ns	0,0634	ns	0,9966	*	0,0288
U-2987MG vs. U-2982MG/SOX2	***	0,0004	*	0,0315	ns	>0,9999
U-2987MG/YFP vs. U-2987MG/SFRP2	ns	0,9966	ns	0,592	ns	0,4995
U-2987MG/YFP vs. U-2982MG	ns	0,9761	ns	0,39	ns	0,1115
U-2987MG/YFP vs. U-2982MG/YFP	ns	0,8089	ns	0,6839	*	0,0315
U-2987MG/YFP vs. U-2982MG/SOX2	*	0,0345	ns	0,8089	ns	>0,9999
U-2987MG/SFRP2 vs. U-2982MG	ns	0,9998	ns	0,9994	ns	0,9482
U-2987MG/SFRP2 vs. U-2982MG/YFP	ns	0,9704	ns	>0,9999	ns	0,7061
U-2987MG/SFRP2 vs. U-2982MG/SOX2	ns	0,1032	ns	0,0689	ns	0,5224
U-2982MG vs. U-2982MG/YFP	ns	0,9952	ns	0,9966	ns	0,9934
U-2982MG vs. U-2982MG/SOX2	ns	0,1738	*	0,0315	ns	0,1204
U-2982MG/YFP vs. U-2982MG/SOX2	ns	0,4111	ns	0,0953	*	0,0345

Supplementary Table S9

Complete list of the significances of the data presented in Figure 4c-e, calculated by one-way ANOVA with Tukey's *post-hoc* tests.

	Circularity		Area		Aspect ratio	
Tukey's multiple comparisons test	Summary	Adjusted P Value	Summary	Adjusted P Value	Summary	Adjusted P Value
U-2987MG vs. U-2987MG/YFP	ns	0,4941	ns	0,8939	ns	>0,9999
U-2987MG vs. U-2987MG/SFRP2	**	0,0016	****	<0,0001	*	0,047
U-2987MG vs. U-2982MG	***	0,0008	ns	0,1643	**	0,0014
U-2987MG vs. U-2982MG/YFP	ns	>0,9999	ns	0,5454	ns	>0,9999
U-2987MG vs. U-2982MG/SOX2	****	<0,0001	****	<0,0001	ns	0,0529
U-2987MG/YFP vs. U-2987MG/SFRP2	ns	0,2631	***	0,0009	*	0,0266
U-2987MG/YFP vs. U-2982MG	****	<0,0001	**	0,0082	**	0,0029
U-2987MG/YFP vs. U-2982MG/YFP	ns	0,418	ns	0,99	ns	>0,9999
U-2987MG/YFP vs. U-2982MG/SOX2	*	0,0175	***	0,0004	*	0,0302
U-2987MG/SFRP2 vs. U-2982MG	****	<0,0001	****	<0,0001	****	<0,0001
U-2987MG/SFRP2 vs. U-2982MG/YFP	***	0,001	**	0,0086	*	0,0306
U-2987MG/SFRP2 vs. U-2982MG/SOX2	ns	0,8929	ns	>0,9999	ns	>0,9999
U-2982MG vs. U-2982MG/YFP	**	0,0012	***	0,0009	**	0,0024
U-2982MG vs. U-2982MG/SOX2	****	<0,0001	****	<0,0001	****	<0,0001
U-2982MG/YFP vs. U-2982MG/SOX2	****	<0,0001	**	0,004	*	0,0347

Supplementary Table S10

Complete list of the significances of the data presented in Figure 5c-d, calculated by one-way ANOVA with Tukey's *post-hoc* tests.

	Vimentin area		GFAP		nestin	
Tukey's multiple comparisons test	Summar y	Adjusted P Value	Summary	Adjusted P Value	Summary	Adjusted P Value
U-2987MG vs. U-2987MG/YFP	ns	0,3443	ns	>0,9999	ns	>0,9999
U-2987MG vs. U-2987MG/SFRP2	ns	0,1398	****	<0,0001	****	<0,0001
U-2987MG vs. U-2982MG	**	0,0019	****	<0,0001	****	<0,0001
U-2987MG vs. U-2982MG/YFP	**	0,0011	****	<0,0001	****	<0,0001
U-2987MG vs. U-2982MG/SOX2	****	<0,0001	ns	>0,9999	ns	>0,9999
U-2987MG/YFP vs. U-2987MG/SFRP2	ns	0,9873	****	<0,0001	****	<0,0001
U-2987MG/YFP vs. U-2982MG	ns	0,0625	****	<0,0001	****	<0,0001
U-2987MG/YFP vs. U-2982MG/YFP	*	0,0335	****	<0,0001	****	<0,0001
U-2987MG/YFP vs. U-2982MG/SOX2	***	0,0006	ns	>0,9999	ns	>0,9999
U-2987MG/SFRP2 vs. U-2982MG	ns	0,1694	ns	>0,9999	****	<0,0001
U-2987MG/SFRP2 vs. U-2982MG/YFP	ns	0,094	ns	>0,9999	ns	0,9953
U-2987MG/SFRP2 vs. U-2982MG/SOX2	**	0,0016	****	<0,0001	****	<0,0001
U-2982MG vs. U-2982MG/YFP	ns	0,9988	ns	>0,9999	***	0,0006
U-2982MG vs. U-2982MG/SOX2	ns	0,1148	****	<0,0001	****	<0,0001
U-2982MG/YFP vs. U-2982MG/SOX2	ns	0,2043	****	<0,0001	****	<0,0001

Supplementary Table S11

Complete list of the significances of the data presented in Figure supplementary 5b, calculated by one-way ANOVA with Tukey's *post-hoc* tests.

	Junctions width	
Tukey's multiple comparisons test	Summary	Adjusted P Value
U-2987MG vs. U-2987MG/YFP	ns	0,6299
U-2987MG vs. U-2987MG/SFRP2	ns	0,0917
U-2987MG vs. U-2982MG	****	<0,0001
U-2987MG vs. U-2982MG/YFP	****	<0,0001
U-2987MG vs. U-2982MG/SOX2	**	0,0018
U-2987MG/YFP vs. U-2987MG/SFRP2	ns	0,8876
U-2987MG/YFP vs. U-2982MG	****	<0,0001
U-2987MG/YFP vs. U-2982MG/YFP	****	<0,0001
U-2987MG/YFP vs. U-2982MG/SOX2	ns	0,1864
U-2987MG/SFRP2 vs. U-2982MG	****	<0,0001
U-2987MG/SFRP2 vs. U-2982MG/YFP	**	0,0033
U-2987MG/SFRP2 vs. U-2982MG/SOX2	ns	0,8149
U-2982MG vs. U-2982MG/YFP	ns	0,4676
U-2982MG vs. U-2982MG/SOX2	***	0,0004
U-2982MG/YFP vs. U-2982MG/SOX2	ns	0,1375

Supplementary Table S12

Complete list of the significances of the data presented in Figure 6c, calculated by one-way ANOVA with Tukey's *post-hoc* tests.

	Invasion	
Tukey's multiple comparisons test	Summary	Adjusted P Value
U-2987MG vs. U-2987MG/SFRP2	***	0,0009
U-2987MG vs. U-2982MG	****	<0,0001
U-2987MG vs. U-2982MG/SOX2	***	0,0005
U-2987MG/SFRP2 vs. U-2982MG	*	0,0183
U-2987MG/SFRP2 vs. U-2982MG/SOX2	ns	0,9314
U-2982MG vs. U-2982MG/SOX2	*	0,0415
